# Global burden of Wilson disease: a comprehensive evidence synthesis

**DOI:** 10.1186/s13023-025-04185-2

**Published:** 2026-01-13

**Authors:** Shan Tang, Wei Hou, Haitian Yu, Yue Wang, Hui Jiang, Zhongping Duan, Sujun Zheng

**Affiliations:** 1 First Department of Hepatology, Beijing YouAn Hospital, Capital Medical University, Beijing, 100069 China; 2 Beijing Institute of Hepatology/Beijing YouAn Hospital, Capital Medical University, Beijing, China

**Keywords:** Wilson disease, ATP7B, Copper metabolism, Prevalence, Prognosis, DALYs, Mental health, Depression, Anxiety, Economic burden

## Abstract

**Background:**

Wilson disease (WD) is a rare inherited copper metabolism disorder with variable prevalence worldwide. Many regions lack reliable data, leading to underestimation of burden. WD presents with hepatic, neurological, and psychiatric symptoms, but mental health impacts and economic costs are often overlooked.

**Methods:**

We systematically searched PubMed up to July 2025 for studies reporting WD prevalence, clinical features, quality of life, or economic burden. Disability-adjusted life years (DALYs) were estimated, and temporal trends were analyzed. Meta-analyses pooled epidemiological and clinical data. Forecast models predicted future prevalence and costs.

**Results:**

A total of 136 studies were included. Prevalence varied, being higher in Southern Europe and parts of Asia, but lower in Northern/Eastern Europe. Rising prevalence was observed in Morocco, South Korea, Spain, and Germany, likely due to improved recognition. Forecasts suggest a gradual global increase through 2030. WD mainly affects young individuals (mean age 19.9 years), with hepatic involvement in 72% and acute liver failure in 31%. Overall mortality was 14%. Mental health complications were frequent, with depression in 47.7% and anxiety in 40%. DALYs have risen globally since the 1970s, with China, the U.S., and Poland projected to carry the heaviest future burden. Economic costs are substantial, particularly in the U.S., where annual hospitalization per patient may exceed USD 60,000.

**Conclusion:**

WD imposes significant clinical, psychological, and economic burdens worldwide. Improved early diagnosis, standardized treatment, and comprehensive care are needed to reduce morbidity, enhance quality of life, and lower healthcare costs.

**Supplementary Information:**

The online version contains supplementary material available at 10.1186/s13023-025-04185-2.

## Background

Wilson Disease (WD) is a rare autosomal recessive disorder of copper metabolism caused by mutations in the *ATP7B* gene. These genetic defects impair hepatic copper transport and biliary excretion, leading to progressive copper accumulation, primarily in the liver and central nervous system [1]. If untreated, copper overload results in irreversible hepatic damage, neuropsychiatric deterioration, and, ultimately, death. WD is one of the few hereditary liver diseases that can be effectively managed if diagnosed early and treated promptly, typically with lifelong copper-chelating agents or zinc salts [2]. However, due to its clinical heterogeneity and often subtle initial symptoms, diagnosis is frequently delayed, particularly in regions lacking access to genetic testing or experienced clinicians.

Despite being described over a century ago, the epidemiological landscape of WD remains poorly defined. Although the global prevalence is estimated at approximately 1 in 30,000 individuals, this figure likely underrepresents the true burden [3]. Higher-than-expected carrier frequencies identified through newborn screening suggest that many cases remain undiagnosed or misdiagnosed [4]. Prevalence estimates vary significantly across countries, with relatively higher rates reported in Spain and Morocco [5, 6]. However, inconsistencies in diagnostic criteria, single-center data, and lack of national registries continue to limit reliable estimation. To date, no systematic effort has synthesized these global data to provide a standardized epidemiological overview.

Clinically, WD presents a broad and complex spectrum of hepatic, neurologic, and psychiatric manifestations. Hepatic symptoms, including elevated transaminases, chronic hepatitis, cirrhosis, and acute liver failure (ALF), are the most common, followed by neurologic symptoms such as tremor, dysarthria, and dystonia [7]. A mixed phenotype of WD also exists, but current evidence regarding the distribution of hepatic, neurologic, and mixed presentations remains inconsistent across studies. Although some data are available on ALF, liver transplantation, and mortality outcomes, these findings are largely derived from isolated cohorts and lack systematic synthesis. Moreover, the long-term prognosis, particularly regarding treatment response, residual disability, and quality of life has not been systematically characterized. There remains a clear need for robust, large-scale analyses to better characterize the full clinical spectrum, disease burden, and prognostic variability of WD.

Beyond physical manifestations, WD also exerts considerable psychological and economic burdens. Depression, anxiety, and cognitive dysfunction frequently affect quality of life, particularly among neurologic cases [8, 9]. In addition, the high cost of lifelong treatment and liver transplantation imposes substantial financial strain on patients and healthcare systems [10]. However, data on these dimensions remain fragmented and regionally limited.

This study aimed to comprehensively characterize the global burden of WD through a systematic review and quantitative synthesis. We summarized worldwide prevalence estimates and meta-analyzed key clinical indicators such as age at onset, hepatic and neurologic involvement, Kayser–Fleischer (KF) rings, urinary copper excretion, ALF, mortality, and liver transplantation. Additionally, we evaluated available data on psychological outcomes and economic impact. By integrating these dimensions, this study provides the first standardized global synthesis of the clinical, psychological, and economic burden of WD, offering evidence to inform early diagnosis, healthcare planning, and equitable management strategies.

## Methods

### Literature search

We performed a comprehensive literature search in PubMed and Embase from database inception to July 2025 without language restrictions. The search strategy, admission criteria and process were shown in the supplementary materials. The study protocol was registered in PROSPERO (Registration ID: CRD420251179894), with registration completed after data extraction and analysis had been conducted.

### Data extraction

Relevant data from all eligible studies were extracted independently by two reviewers (ST and WH) using a pre-designed standardized spreadsheet. Any discrepancies were resolved by discussion with a third reviewer (SJZ) to ensure accuracy and consistency. Extracted data were categorized into four domains: (1) clinical and demographic characteristics, (2) epidemiological indicators, (3) economic burden, and (4) quality of life and mental health. Extracted variables included study details (first author, year of publication, country, study type, and study period), patient demographics (age, sex, disease subtype), clinical characteristics (Kayser-Fleischer rings, ceruloplasmin levels, hemolytic anemia, urinary copper excretion, oral treatment), and clinical outcomes (ALF, mortality, liver transplantation, and follow-up duration). For epidemiological analysis, data on annual incidence, sex-stratified population sizes, prevalence, allele frequency, carrier frequency, and family history were also collected. For studies reporting patient-reported outcomes, data such as SF-12 scores, PHQ-9 scores, EQ-VAS scores, and other relevant measures were extracted.

### Prevalence pooling approach

We performed a formal meta-analysis using inverse-variance weighted random-effects modeling. For each study, the point prevalence (per 100,000 population) and its standard error were extracted. Pooled national prevalence estimates were then derived using the DerSimonian-Laird random-effects model, and 95% confidence intervals for pooled prevalence were computed using the Clopper-Pearson exact method. For visualization, countries were categorized into five prevalence levels based on pooled estimates: 1) Very high: ≥10 per 100,000; 2)High: 5–9.99 per 100,000; 3) Medium: 1–4.99 per 100,000; 4)Low: 0.5–0.99 per 100,000; 5)Very low: <0.5 per 100,000.

### Disease burden estimation

To estimate disease burden, disability-adjusted life years (DALYs) were calculated where data were sufficient. DALYs were determined according to Global Burden of Disease (GBD) [11] methodology by summing years of life lost (YLL) due to premature mortality and years lived with disability (YLD). YLLs were calculated based on reported age at death and reference life expectancy, while YLDs were estimated using disease duration and standardized disability weights from GBD studies [12] (Supplementary Materials, Supplementary Table 1).

### Trend analysis

Temporal trends in prevalence were analyzed using linear regression and the Mann-Kendall trend test. Linear regression models used calendar year as the independent variable, while the Mann-Kendall test, a non-parametric method, was used to detect monotonic trends over time. These analyses were performed to evaluate whether WD prevalence has changed significantly in recent decades.

### Time series forecasting

For indicators such as prevalence and economic burden, where annual data were consistently available, autoregressive integrated moving average (ARIMA) models were applied to forecast future trends. Forecasts were generated for a 10-year horizon, and model performance was evaluated through residual diagnostics. Forecasts were visualized with confidence intervals and plots comparing observed versus predicted trends. For prevalence forecasts, outputs included heatmaps to visualize temporal trends and world maps to display geographic distribution patterns.

### Quality assessment

The methodological quality was evaluated using the Joanna Briggs Institute (JBI) Critical Appraisal Checklist [13]. Nine domains were assessed. Each item was rated as “Yes” or “No” according to the published methodology.

### Publication bias assessment

Publication bias was evaluated following recommendations from the Cochrane Handbook for Systematic Reviews of Interventions [14]. Funnel plots were visually inspected for asymmetry. To ensure sufficient statistical power, Egger’s regression test was applied only to outcomes informed by ten or more studies, using the standard error as the precision measure. For outcomes with three to nine studies, only descriptive visualization of funnel-plot patterns was performed. Funnel plots were generated separately for clinical outcomes and quality-of-life outcomes. A p-value < 0.05 from Egger’s test (for outcomes with ≥ 10 studies) was considered indicative of potential publication bias.

### Statistical analysis

Descriptive statistics were used to summarize the characteristics of the included studies and populations. For variables reported across multiple studies, meta-analyses were performed to estimate pooled means or proportions. Continuous variables (e.g., age, quality of life scores) were pooled using the inverse variance method, while binary variables (e.g., KF ring positivity) were pooled to estimate prevalence. Between-study heterogeneity was described using the I^2^ statistic. A random-effects model was used as the conceptual default, given the expected variability in study design, populations, and follow-up duration across the included studies. Meta-analysis results are presented as forest plots.To explore sources of heterogeneity, we conducted mixed-effects meta-regression for the four primary outcomes. Event proportions were logit-transformed, and random-effects meta-regression was performed using weighted least squares, with weights equal to 1/(vi + τ²), where vi represents within-study variance. Predefined study-level covariates included publication year, log-transformed sample size (logN), geographic region, and study design. Sensitivity analyses were conducted by excluding outliers to assess model robustness. A two-sided *p* < 0.05 was considered statistically significant. All statistical analyses were conducted using R software (version 4.3.0), further details are provided in the Supplementary Materials.

## Results

### Search results

**Fig. 1:**
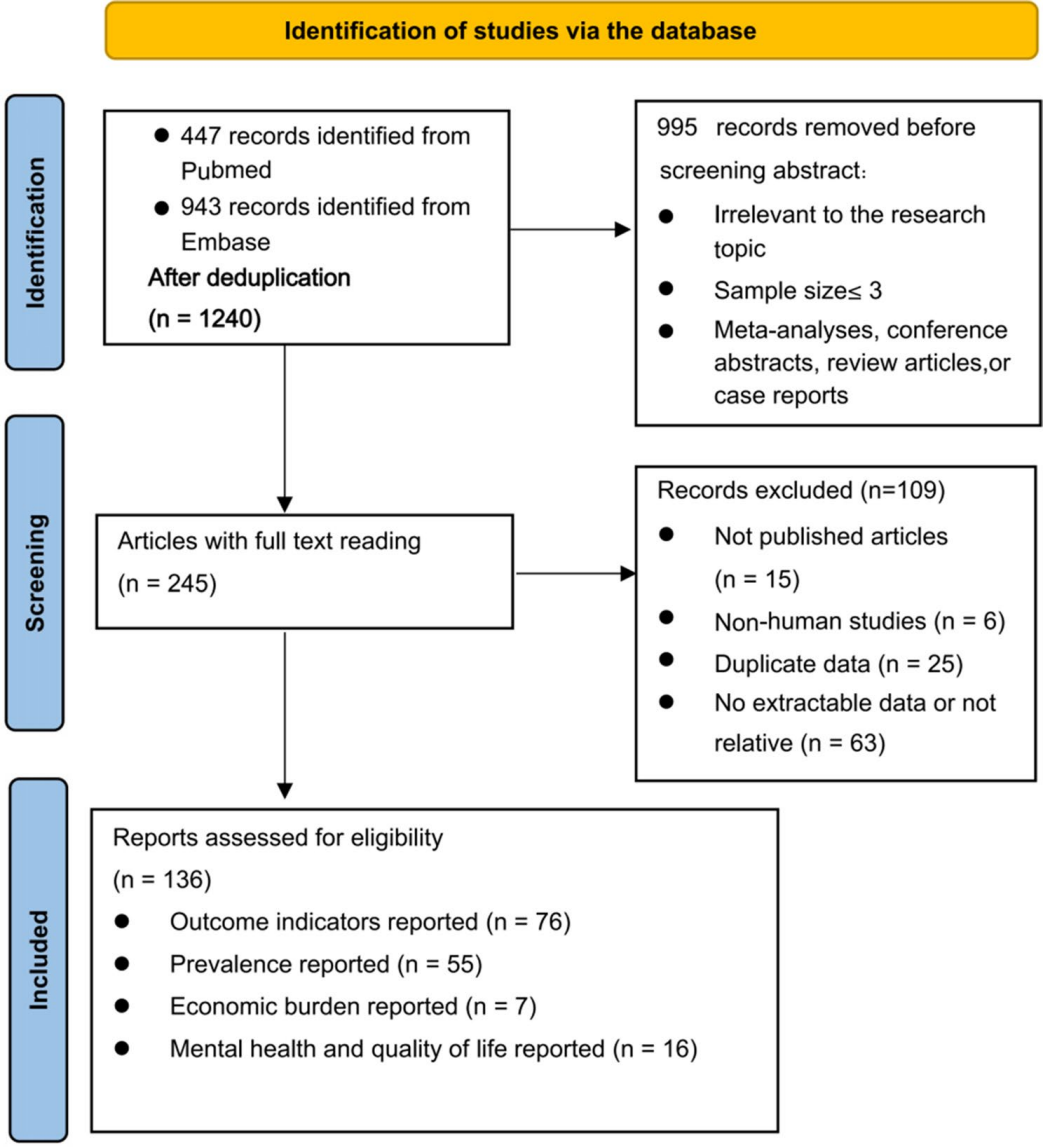
Flow chart summarizing the literature search and selection process

A total of 1390 records were initially identified through the PubMed and Embase database. of which, 150 studies were excluded as duplicates. After screening the titles and abstracts, 995 records were excluded for the following reasons: irrelevance to the research topic, small sample size (≤ 3), or being review articles, meta-analyses, conference abstracts or case reports. The remaining 245 articles were subjected to full-text review. Among these, 109 articles were excluded due to the following reasons: no extractable data (*n* = 63), duplicate data (*n* = 25), non-human studies (*n* = 6), and inability to obtain the full text (*n* = 15). Finally, 136 studies were included in this systematic review. Of these, 76 studies reported clinical outcomes of WD patients, 55 reported prevalence data, 7 addressed the economic burden, and 16 studies provided data on mental health and quality of life (Fig. 1).

### Global trends in the prevalence of WD

**Fig. 2:**
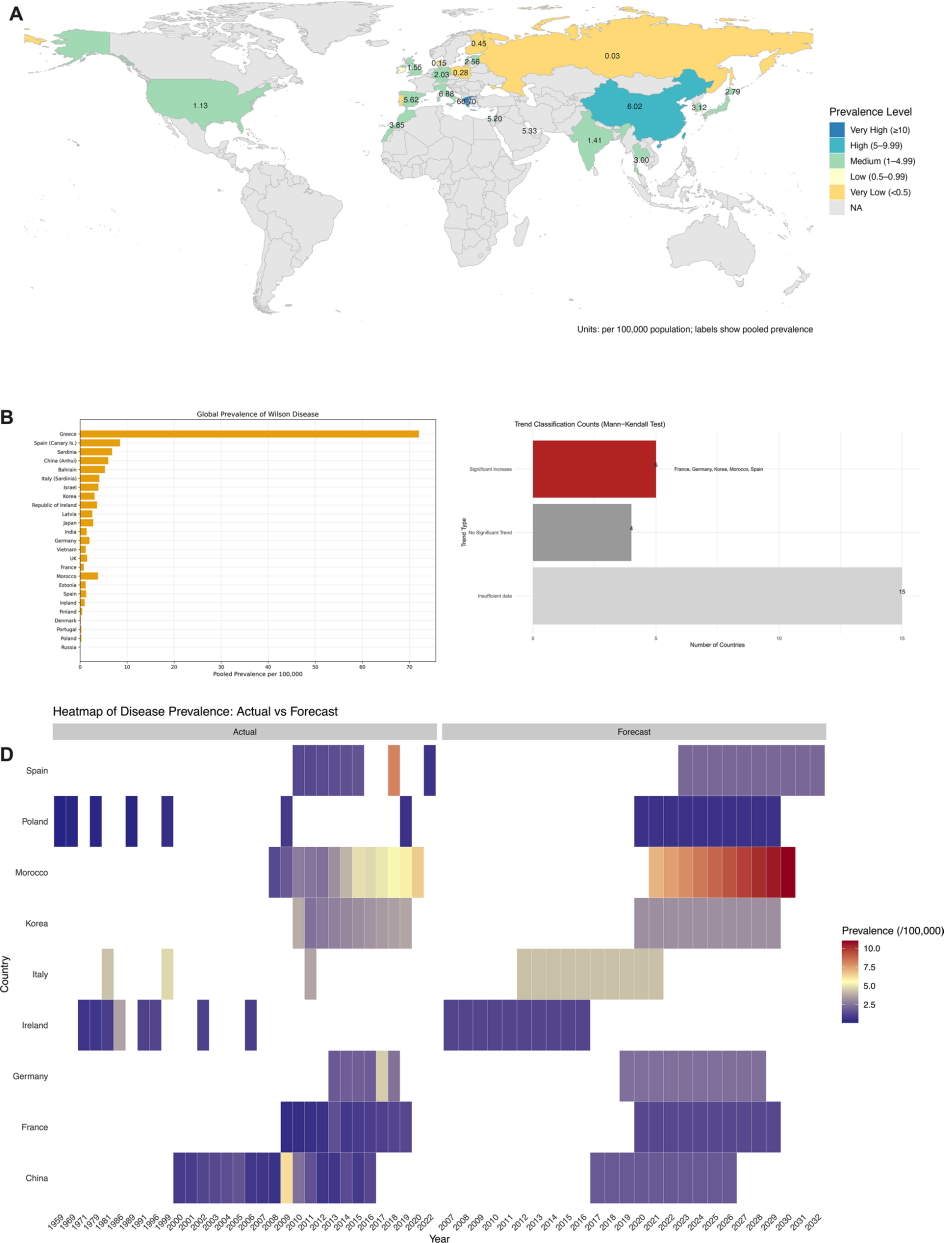
Epidemiological summary of global prevalence patterns of WD based on pooled data and forecasting models. (**A**) Average prevalence of WD by country, categorized by prevalence level. Countries were grouped into five prevalence categories (very low, low, medium, high, very high) and visualized by color on the world map. (**B**) Ranked bar plot of average WD prevalence per 100,000 population by country. (**C**) Trend Classification by Mann-Kendall Test. Bar plot showing the number of countries classified by trend type using the Mann-Kendall test. Trends are categorized into significant increase, no significant trend, and insufficient data. (**D**) Heatmap of actual versus forecasted prevalence trends in selected countries. The left panel displays actual historical data, while the right panel presents ARIMA-model-based forecasts. Color scales reflect prevalence magnitude

In total, 55 studies [5, 15–68] covering 32 distinct countries or regions were included in the supplementary data synthesis (Supplementary Table 2). Covering Europe, Asia, and the Americas. The highest pooled estimate was reported in a localized, founder-effect cohort from Greece (66.7 per 100,000), which reflects a genetically isolated population rather than a nationwide prevalence level. Several additional high-prevalence clusters were identified, including Sardinia (6.88), China–Anhui (6.02), Spain–Canary Islands (5.62), Bahrain (5.33) and Israel (5.20)—all falling within the 5–9.99 per 100,000 range. At the national level, most countries demonstrated moderate prevalence (1–4.99 per 100,000), including Italy (4.13), Morocco (3.85), Ireland (3.60), South Korea (3.12), Thailand (3.00), Japan (2.79), Latvia (2.56), Germany (2.03), the United Kingdom (1.55), India (1.41), Spain (1.27), Estonia (1.24), and the United States (1.13). Lower prevalence levels (0.5–0.99 per 100,000) were observed in Ireland (0.97) and France (0.80). The very lowest pooled estimates (< 0.5) were noted in Finland (0.45), Poland (0.28), Portugal (0.27), Denmark (0.15), and Russia (0.03) (Fig. 2A–B, Supplementary Fig. 1). Mann–Kendall tests indicated significant upward trends in France, Germany, South Korea, Morocco, and Spain, likely reflecting improved diagnostics. Northern and Eastern Europe and parts of Asia generally showed low and stable prevalence (Fig. 2C). ARIMA forecasts predict modest annual increases (~ 2–3%) through 2030. Morocco may reach ~ 11.0 by 2030; Spain may stabilize near 2.16 by 2032; China around 1.76 by 2026; Poland remains ~ 0.59 through 2029 (Fig. 2D).

### Clinical features and prognosis of WD

A total of 76 studies [5, 19, 26, 31, 37, 41, 45, 50, 54, 55, 57, 58, 60, 69–131] including over 35,000 patients with WD were analyzed, ranging from small single-center cohorts (e.g., Egypt, *n* = 77; Pakistan, *n* = 48; Morocco, *n* = 226) to large population-based registries in Germany, China, South Korea, and the U.S. (1,000–5,000 cases). These studies reported substantial variability in age at diagnosis, sex distribution, clinical phenotypes (hepatic vs. neurological), KF ring positivity, ceruloplasmin levels, urinary copper excretion, and treatment regimens, with some detailing treatment outcomes, ALF, mortality, and liver transplantation (Supplementary Table 3).

Meta-analysis showed WD predominantly affects young individuals, with a pooled mean age of 19.93 years (95% CI: 15.74–24.13; I^2^ = 99.9%, k = 26), and a slight male predominance (53%, 95% CI: 52%–55%; I^2^ = 36.1%, k = 49). Hepatic involvement was most common (72%, 95% CI: 61%–79%; I^2^ = 95.0%, k = 53), while neurological symptoms occurred in 28% (95% CI: 22%–36%; I^2^ = 88.2%, k = 44). The KF ring was highly sensitive, positive in 88% (95% CI: 84%–91%; I^2^ = 64.5%, k = 37). Laboratory findings included markedly elevated 24-hour urinary copper (pooled mean 739.03 µg, 95% CI: 461.33–1016.74; I^2^ = 99.0%, k = 15) and low ceruloplasmin prevalence (24%, 95% CI: 13%–40%; I^2^ = 88.8%, k = 30). Hemolytic anemia occurred in 16% (95% CI: 9%–26%; I^2^ = 91.4%, k = 23) (Supplementary Figs. 2–9).

**Fig. 3:**
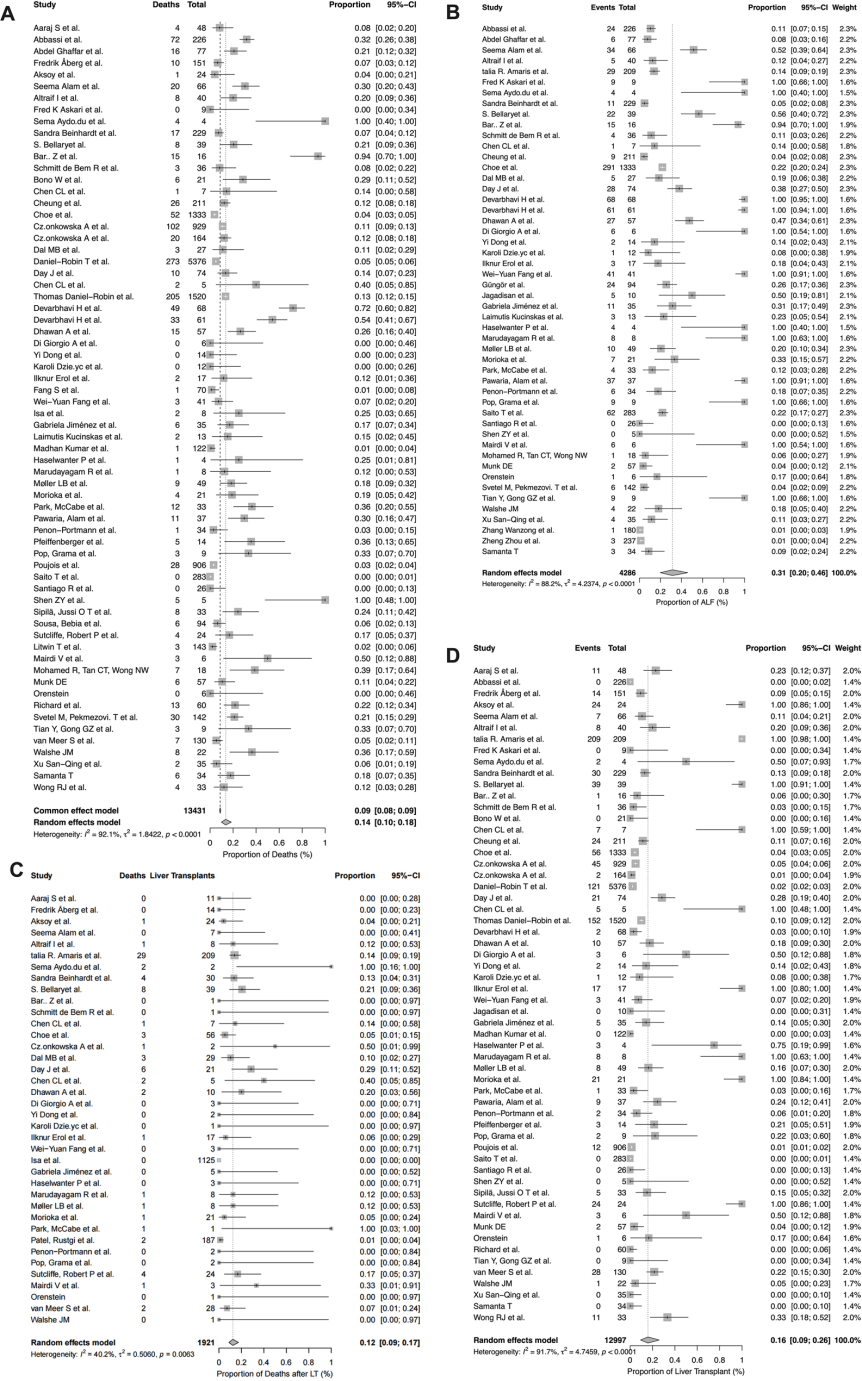
Forest plots summarizing the pooled proportions of key clinical outcomes in patients with WD across included studies. (**A**) Proportion of deaths among WD patients. (**B**) Proportion of WD patients presenting with ALF. (**C**) Proportion of deaths after liver transplantation in WD patients. (**D**) Proportion of WD patients undergoing liver transplantation. Note: Pooled proportions and 95% confidence intervals (CIs) were estimated using random-effects models. The weight of each study is indicated. Heterogeneity statistics (I², χ^²^ and p-value) are provided for each analysis

Clinical outcomes were concerning. The pooled proportion of deaths among WD patients was 14% (95% CI: 10%–18%), with substantial heterogeneity (I² = 92.1%, *p* < 0.001), based on 53 studies (Fig. 3A). The mean follow-up duration among these mortality studies was 7.3 ± 6.7 years (median 6.3 years). The pooled proportion of ALF was 31% (95% CI 20%–46%), again with marked heterogeneity (I² = 88.2%, *p* < 0.001), derived from 47 studies (Fig. 3B), derived from 47 studies. Among transplanted patients, the post-transplant mortality was 12% (95% CI: 9%–17%), showing moderate heterogeneity (I² = 40.2%, *p* = 0.006), from 39 studies (Fig. 3C), and the proportion of WD patients undergoing liver transplantation was 16% (95% CI: 9%–26%), with high heterogeneity (I² = 91.7%, *p* < 0.001), based on 41 studies (Fig. 3D).

To explore potential sources of heterogeneity, mixed-effects meta-regression was performed. Meta-regression revealed that logN was inversely associated with event rates for ALF (β=-0.664, *p* = 0.002), deaths (β=-0.366, *p* = 0.042), and liver transplantation (β=-0.620, *p* = 0.001), suggesting small-study or reporting effects. Publication year showed a significant negative association with overall mortality (β=-0.039, *p* = 0.004) and post-transplant death (β=-0.082, *p* = 0.019), implying improved survival in more recent studies. No consistent effects were observed for geographic region or study design, except that cohort-type studies tended to report higher post-transplant mortality (β=+1.633, *p* = 0.038)(Supplementary Table 4, Supplementary Fig. 10). Sequential leave-one-out analysis and exclusion of small-sample studies (*n* < 20) did not materially alter pooled proportions (variation < 2%), and fixed-effects sensitivity models produced consistent results(Supplementary Table 5; Supplementary Fig. 11).

### Global disease burden of WD (DALYs)

**Fig. 4:**
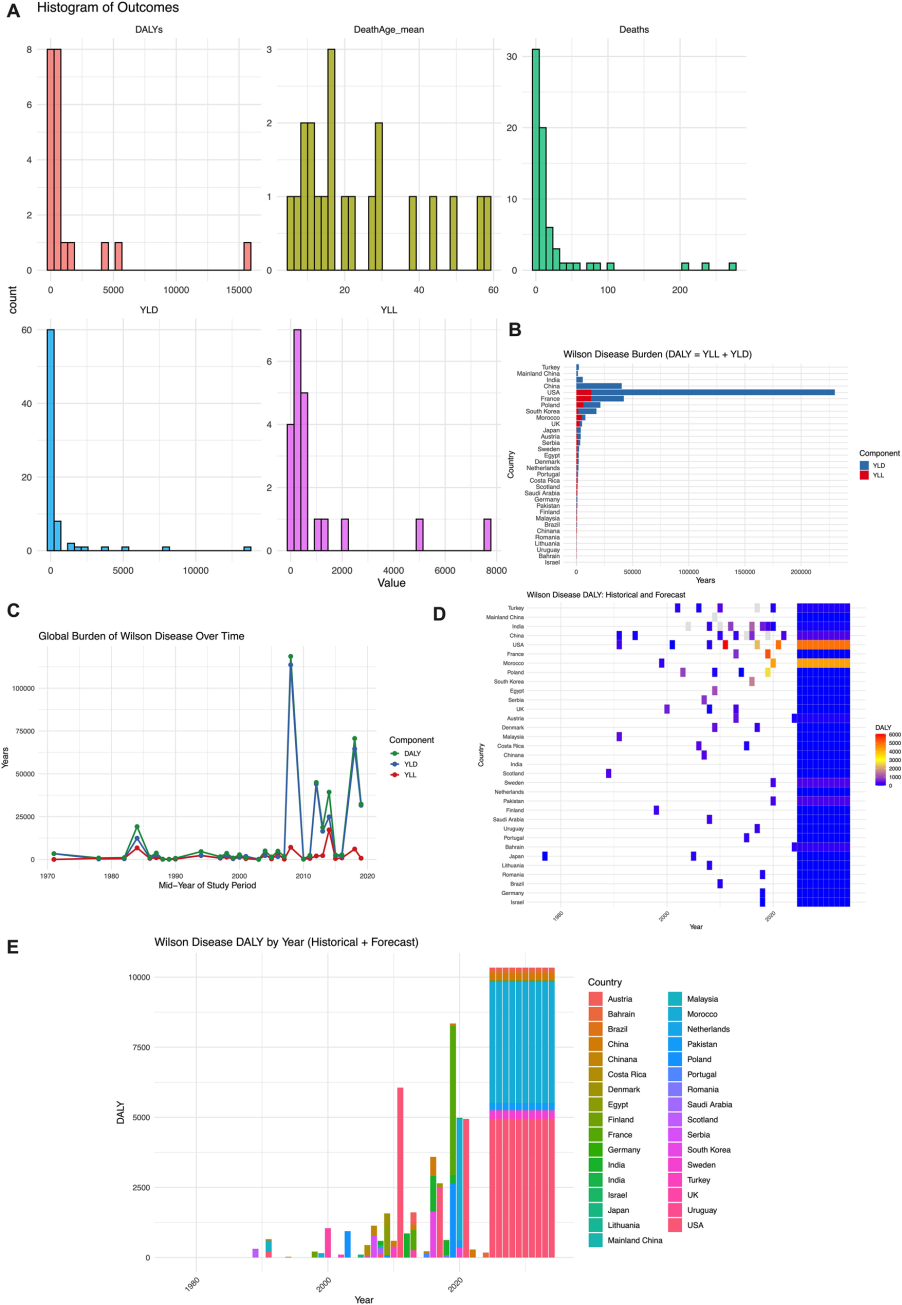
Global burden of WD in disability-adjusted life years (DALYs), years of life lost (YLL), years lived with disability (YLD), and deaths across included studies. (**A**) Histograms summarizing the distribution of key outcome indicators across countries, including DALYs, YLD, YLL, number of deaths, and mean age at death. (**B**) Bar plot of total DALYs stratified by country, with DALY components (YLD vs. YLL) indicated by color. (**C**) Global temporal trends in DALYs, YLD, and YLL related to WD from the 1970s to 2020. (**D**) Heatmap showing historical data and ARIMA-model-based projections of DALYs by country and year. Color gradients reflect the magnitude of DALYs, highlighting countries expected to experience sustained or increasing disease burden in future decades. (**E**) Stacked bar plot of historical and forecasted DALYs by country from 1970 to 2050. Countries such as China, India, the United States, Morocco, and Poland are projected to have the highest future disease burden. Note: All estimates are based on aggregated or forecasted data using available national reports, epidemiological studies, and modeling approaches. DALYs are calculated as the sum of YLD and YLL

The global burden of WD, measured by DALYs, Years of Life Lost (YLL), Years Lived with Disability (YLD), deaths, and mean age at death, exhibits marked heterogeneity. Most countries report relatively low DALYs (< 5,000), while a few regions carry disproportionately high burdens. Similar patterns are seen for YLL and YLD, indicating that severe disease burden is concentrated in specific populations. Deaths and mean age at death also vary widely, reflecting differences in healthcare systems and diagnostic practices (Fig. 4A). To further explore these disparities, we compared country-level DALY composition and magnitude. As shown in Fig. 4B, the United States exhibited the highest burden of WD, reaching 229,967 years (person-years). The next highest burdens were observed in China (~ 36,000 years), France (~ 42,000 years), Poland (~ 21,000 years), and South Korea (~ 18,000 years). India showed a moderate level (~ 11,000 years), while most other countries, such as Japan, Austria, Denmark, the Netherlands, Portugal, and Saudi Arabia, had relatively low overall burdens (< 5,000 years). Temporal trends show a generally upward trajectory in DALYs over the past five decades, rising from ~ 400 in the 1970s to ~ 2,000 by 2020. Decadal averages increased from 492.5 in the 1980s to 598.1 in the 1990s, 1,042.6 in the 2000s, and 1,386 in the 2010s, likely reflecting improved diagnostic awareness, population growth, and aging demographics, alongside fluctuations in reporting quality (Fig. 4C). Country-level projections highlight China, India, the United States, Morocco, and Poland as regions with substantial expected increases in DALYs, driven by demographic shifts, healthcare disparities, and under-recognized disease burden (Fig. 4D). China is projected to have one of the highest burdens due to its large population and uneven healthcare resources, while India’s burden is expected to rise sharply after 2030. Forecasts indicate that global DALYs will continue rising, emphasizing the growing healthcare demand associated with WD (Fig. 4E).

### Mental health burden and quality of life in patients with WD

**Fig. 5:**
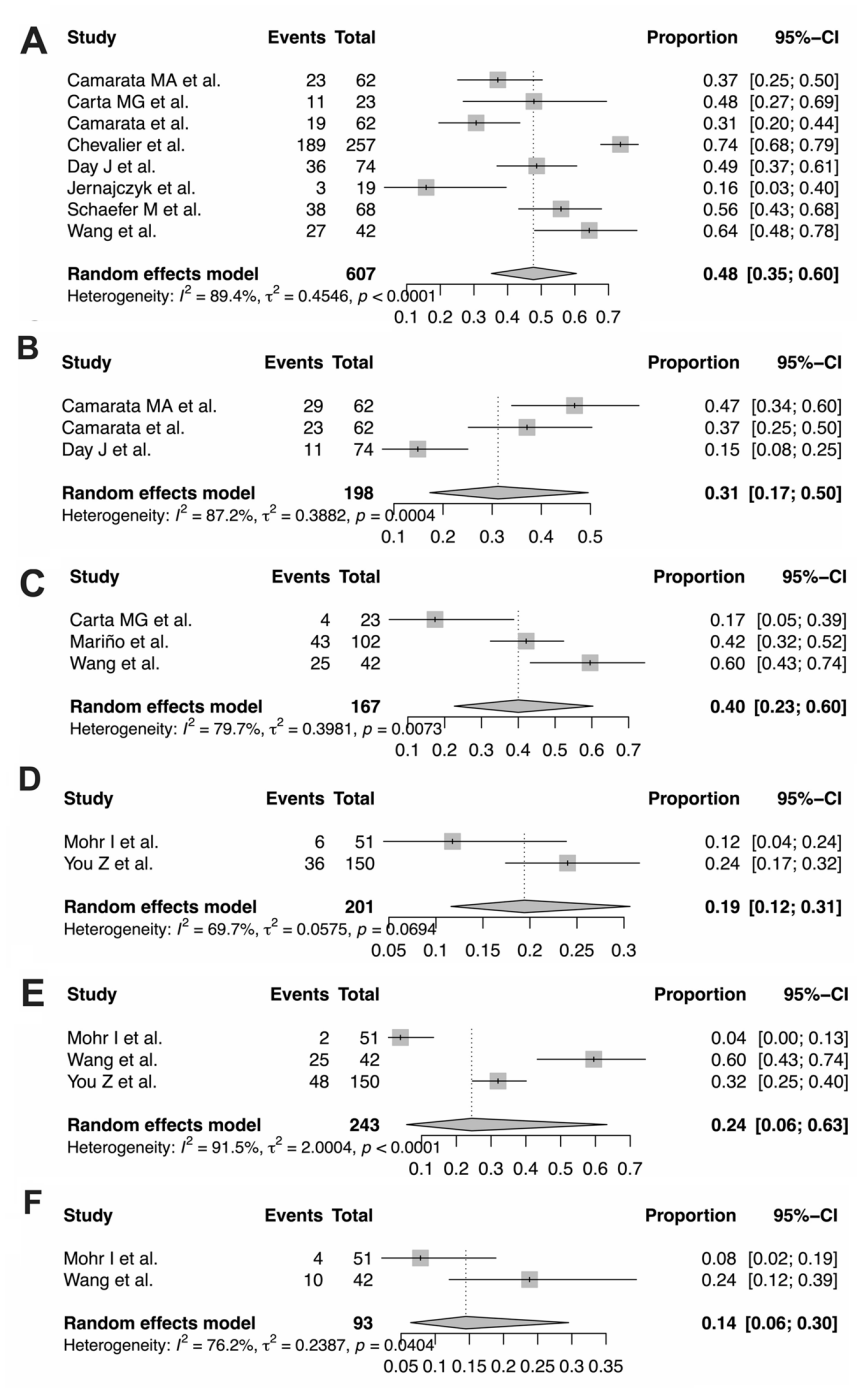
Forest plots summarizing the pooled estimates of neuropsychiatric symptoms, quality of life, and related outcomes in patients with WD across included studies. (**A**) Prevalence of depressive symptoms. (**B**) Prevalence of cognitive impairment. (**C**) Prevalence of anxiety. (**D**) Prevalence of fatigue. (**E**) Prevalence of sleep disorders. (**F**) Prevalence of daytime sleepiness. Note: Pooled proportions and 95% confidence intervals (CIs) were estimated using random-effects models for prevalence outcomes. The weight of each study is indicated. Heterogeneity statistics (I², χ², and p-value) are provided for each analysis

In addition to epidemiological and clinical characteristics, 16 studies [115, 132–146] assessed quality of life (QOL), psychiatric comorbidities, and neurocognitive outcomes in approximately 1,135 patients with WD, with sample sizes from 19 to 257 per study. Mental and physical health were evaluated using standardized tools, including SF-12 (Physical and Mental Component Scores), PHQ-9, and EQ-VAS. Psychiatric conditions assessed included depression, anxiety, bipolar disorder, panic disorder, cognitive impairment, fatigue, sleep disturbances, and daytime sleepiness (Supplementary Table 6). Depressive symptoms affected 47.7% of patients (95% CI: 35.2–60.5; I^2^ = 91.6%, k = 6), anxiety 40.0% (95% CI: 22.6–60.3; I^2^ = 93.8%, k = 3), and cognitive impairment 31.2% (95% CI: 17.3–49.6; I^2^ = 87.7%, k = 3). Fatigue was observed in 19.4% (95% CI: 9.6–34.8; I^2^ = 92.3%, k = 2), sleep disorders in 24.4% (95% CI: 9.8–49.6; I^2^ = 79.5%, k = 2), and daytime sleepiness in 14.5% (95% CI: 9.6–20.8; I^2^ = 78.6%, k = 2), respectively (Fig. 5). Bipolar disorder and panic disorder were less frequently reported, occurring in 14.3% and 8.2% of patients. Health-related QOL was reduced, with SF-12 mental and physical scores of 52.0 and 54.6, EQ-VAS 77.8, and PHQ-9 4.4, indicating mild to moderate depressive risk. High heterogeneity (I² = 70–92%) reflected differences among studies (Supplementary Fig. 12).

### Economic burden and future projections of WD

In addition to epidemiological, clinical, and quality of life data, 7 studies [48, 54, 118, 140, 147–149] assessed the economic burden of WD in over 37,000 patients across diverse healthcare settings, reporting hospitalization, liver transplant, outpatient, sick leave, and other direct or indirect costs (Supplementary Table 7).

**Fig. 6:**
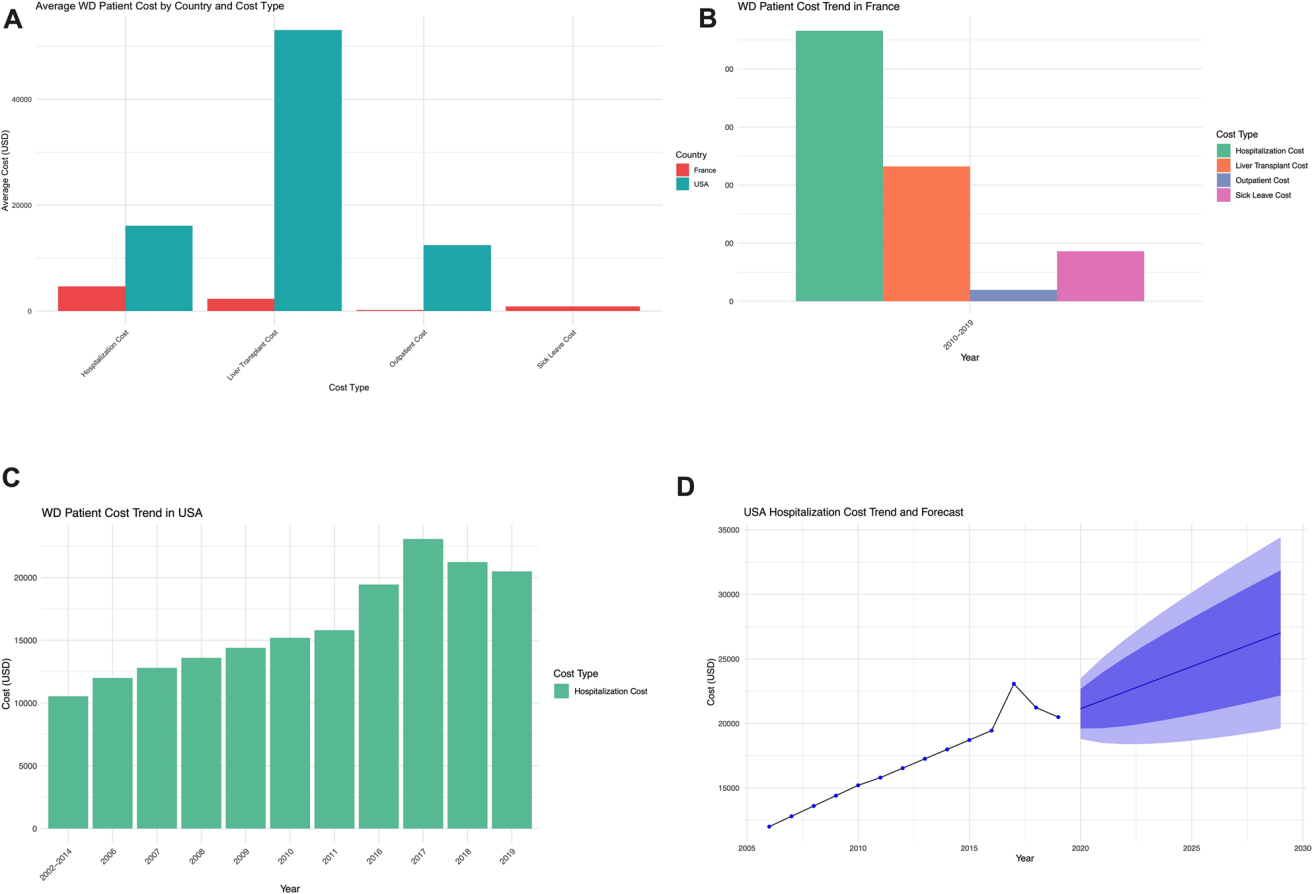
Economic burden of WD in the United States and France. (**A**) Comparison of average healthcare costs for WD patients between the United States and France, categorized by cost type. This bar chart presents average costs for WD patients in the United States and France, categorized by cost type, including hospitalization, liver transplantation, outpatient care, and sick leave. Bar heights reflect the magnitude of costs, with different colors representing different cost categories. (**B**) Breakdown of healthcare costs for WD patients in France by cost type. This panel illustrates the composition of healthcare costs for WD patients in France, detailing hospitalization, liver transplantation, outpatient care, and sick leave. (**C**) Annual trends in hospitalization costs for WD patients in the United States (2002–2019). This figure shows the annual hospitalization costs for WD patients in the United States from 2002 to 2019, using bar charts to depict year-by-year fluctuations and the overall rising trend. (**D**) Hospitalization cost trends and forecasts for WD patients in the United States. This panel illustrates historical (2005–2019) and forecasted (2020–2030) hospitalization costs for WD patients in the United States. The line graph displays observed data and ARIMA-based projections, with shaded areas indicating the uncertainty range. A continued upward trend in hospitalization costs is predicted

Significant inter-country differences were observed. In the United States, the mean annual per-patient hospitalization cost ranged widely from USD 10,543 to 61,648, while the mean cost per liver transplantation reached approximately USD 53,054 per procedure. In contrast, France demonstrated a more balanced cost distribution, with mean annual per-patient hospitalization costs of USD 4,658, transplantation costs of USD 2,322, outpatient costs of USD 195, and sick-leave costs of USD 860. While hospitalization accounted for the majority of total expenses in the United States, French data reflected a broader inclusion of indirect and non-medical costs (Fig. 6A–C).

Temporal trends further showed that in the U.S., annual per-patient hospitalization costs increased from approximately USD 10,543 in 2002 to over USD 30,000 in 2019, with projected annual growth rates of 5–8% through 2030, driven by rising medical costs, improved diagnostic awareness, and greater utilization of advanced treatment modalities (Fig. 6D).

### Study quality and publication bias assessment

As summarized in Supplementary Table 8, among the 76 studies reporting clinical outcomes, 29 (38.2%) were rated as high quality, 41 (53.9%) as moderate quality, and 6 (7.9%) as low quality according to the JBI Critical Appraisal Checklist.

Given the heterogeneity in study quality, potential publication bias was further evaluated. For primary clinical outcomes, visual inspection of the funnel plots (Supplementary Fig. 13) revealed clear asymmetry across several endpoints. Egger’s regression confirmed statistically significant asymmetry for acute liver failure (*p* = 6.9 × 10^− 10^), overall mortality (*p* = 1.1 × 10^− 22^), and liver transplantation (*p* = 1.1 × 10^− 18^), indicating possible small-study or reporting effects. Post-transplant death (*p* = 0.0116) showed a weaker but notable signal of bias (Supplementary Table 9). For quality-of-life outcomes, the funnel plots (Supplementary Fig. 14) displayed mild to moderate asymmetry, particularly for SF-12 Mental and Physical QOL domains, consistent with Egger’s regression results (*p* = 0.0009 and *p* = 0.0001, respectively). PHQ-9 (*p* = 0.0012) and depression (*p* = 0.0202) also showed indications of possible publication bias, while anxiety (*p* = 0.8532), cognitive impairment (*p* = 0.1246), and sleep disorder (*p* = 0.9502) exhibited largely symmetrical distributions without significant asymmetry (Supplementary Table 9).

## Discussion

In this comprehensive analysis, we evaluated the epidemiological trends, clinical characteristics, disease outcomes, mental health burden and economic burden of WD from a global perspective. Our findings offer an updated and holistic view of the disease profile, highlighting several critical issues in WD diagnosis, management, and prognosis.

Firstly, our analysis of global epidemiological data confirms substantial geographic variability in the reported prevalence of WD. The highest prevalence persists in southern European regions such as Greece, Sardinia, and Canary Islands likely reflecting founder effects and regional genetic clusters. Conversely, lower prevalence rates in Asia and Eastern Europe likely influenced by differences in case ascertainment and availability of genetic testing rather than true disease frequency. Notably, recent upward trends observed in countries such as Germany and South Korea may reflect enhanced diagnostic capacity and clinical awareness, though causality cannot be confirmed [58, 61]. Mann-Kendall trend analysis and ARIMA projections indicated modest increases in prevalence, these predictions should be interpreted cautiously given the heterogeneity of available data and the limited representation of several world regions [150]. Overall, the marked geographic variability underscores persistent gaps in surveillance, particularly in underrepresented areas, and highlights the need for harmonized diagnostic pathways and national or regional WD registries.

Consistent with previous literature [151, 152], our results confirm that WD predominantly affects young individuals, with a pooled mean age below 20 years, and exhibits a slight male predominance. The clinical manifestations remain centered on hepatic involvement, affecting over 70% of patients, while neurological symptoms account for roughly 30%. These findings reinforce the classical phenotypic spectrum of WD, where liver involvement typically presents earlier, whereas neurological symptoms often emerge later, especially in patients with delayed diagnosis. However, the pooled prevalence of KF rings (88%) was notably higher than that typically reported among patients with isolated hepatic presentations, where positivity has historically 50% [153]. This likely reflects the case mix of included studies, many of which enrolled patients with mixed or neurologic symptoms, where KF rings positivity approaches 80–100%. In addition, most studies originated from tertiary-care centers, where patients often present with more advanced disease. Regional genetic clusters in high-prevalence areas may also contribute to this pattern. These factors together explain the higher pooled KF rings estimate observed in our analysis. These considerations suggest that while KF ring remains an important diagnostic marker, its pooled prevalence in this review should not be generalized to all hepatic-only WD populations.

Besides, our analysis also reveals a concerning prognosis despite standard therapies. Information on therapeutic regimens was available for most included studies, showing that D-penicillamine remained the most commonly used first-line chelator in the included studies, followed by zinc salts administered as monotherapy or in sequential and combination regimens, treatment practice varied considerably across cohorts. Treatment coverage across the included studies averaged approximately 85%. Poor adherence, treatment discontinuation, or delayed initiation of copper-lowering therapy were frequently reported. These factors may partially explain why nearly one-third of patients progressed to ALF and why overall mortality remained substantial at 14%, underscoring the ongoing challenges in long-term disease management despite the availability of effective therapies. Post-transplantation mortality reached 31%, underscoring the complexity of disease management even after surgical intervention. These findings suggest that while access to established copper-lowering agents is relatively widespread, disparities persist in treatment availability, initiation timing, adherence, and long-term follow-up, which may contribute to the observed variability in survival outcomes. Collectively, these challenges emphasize the need for standardized diagnostic and therapeutic pathways, early intervention strategies, and more precise prognostic markers to optimize long-term disease control and improve global outcomes in WD.

The analysis of DALYs reveals a steadily increasing global burden of WD over the past five decades, with overall DALYs approximately five-fold higher than in the 1970s. This upward trajectory is consistent with improved case identification and increased survival, both of which contribute to a growing cumulative burden. Interestingly, high DALYs values were not confined to developed countries but were also prominent in middle-income regions such as Morocco and Poland, potentially indicating disparities in healthcare access, delayed diagnoses, and variable treatment quality. Previous studies have rarely quantified WD’s global burden in DALYs; thus, our findings fill an important gap by highlighting the growing public health significance of WD despite its classification as a rare disease. The accelerated rise in DALYs since the early 2000s also parallels broader improvements in diagnostic capacity and awareness reported in contemporary WD studies. These observations underscore the importance of strengthening early detection and sustained long-term management to reduce preventable disability. Given the increasing DALY burden and associated economic impact, integrating WD into national rare disease planning may help support more equitable healthcare delivery and resource allocation. Furthermore, international collaboration and data sharing are essential to optimize healthcare delivery and promote equitable access worldwide.

Importantly, alongside the increasing epidemiological and disease burden, our meta-analysis also emphasizes the substantial mental health challenges faced by WD patients. Nearly half experience depressive symptoms, while anxiety and cognitive impairments are also highly prevalent, significantly exceeding rates observed in the general population. These findings align with neurobiological evidence linking copper deposition in the basal ganglia and limbic system to neuropsychiatric manifestations. Additionally, sleep disorders, fatigue, and daytime somnolence contribute to the overall morbidity but are frequently underrecognized in clinical practice. Moreover, quality of life assessments (SF-12 and EQ-VAS) reveal significant reductions in both mental and physical domains compared to normative populations, consistent with prior studies that highlight the chronic and multifaceted burden of WD. Although there is substantial heterogeneity in mental health data, these results collectively underscore the urgent need for integrated care models that address both hepatic and neuropsychiatric components of the disease. Routine psychological screening and timely, appropriate interventions should be incorporated into standard WD management to improve treatment adherence, functional outcomes, and overall prognosis. Moreover, the current lack of structured mental health support represents a critical gap in patient care. Given the high prevalence of depression, anxiety, and cognitive impairments among individuals with WD, it is essential to integrate comprehensive psychological assessment and targeted interventions into routine clinical guidelines. Multidisciplinary collaboration and dedicated patient support programs are vital to enhancing quality of life and promoting sustained treatment adherence.

Beyond mental health challenges, our analysis indicates marked variability in the economic burden of WD across countries. The United States demonstrated substantially higher annual per-patient costs, largely attributable to hospitalization and liver transplantation, consistent with previous reports describing elevated expenditures in settings with complex healthcare financing systems. In contrast, France exhibited a more balanced distribution of direct and indirect costs, including productivity loss and sick leave, highlighting the broader societal impact of WD. Forecasting analyses suggest that hospitalization-related costs in the United States may continue to rise through 2030, reflecting increasing healthcare utilization associated with WD. These observations align with economic evaluations of other rare diseases, in which late-stage complications account for a disproportionate share of total expenditures [154]. Strengthening international cost surveillance systems and promoting cost-effective strategies such as earlier diagnosis and broader access to genetic testing may help mitigate future financial burden. Additionally, optimizing care pathways to prevent acute liver failure and reduce hospitalization frequency represents an important priority for improving both clinical outcomes and economic efficiency.

Funnel plots and Egger’s regression tests demonstrated statistically significant asymmetry across several primary outcomes, including acute liver failure, overall mortality, liver transplantation, and post-transplant death (all *p* < 0.05), suggesting the presence of publication bias. Beyond conventional “positive-results bias,” several contextual factors likely contributed to this asymmetry. Earlier publications and tertiary-care center cohorts disproportionately represented patients with severe disease, leading to inflated event rates. In addition, structural differences across study populations, such as the inclusion of pediatric versus adult cohorts, and variations in therapeutic eras, including access to chelators, zinc, or trientine, may have introduced systematic imbalances. Moreover, Egger’s test is inherently sensitive to heterogeneity, particularly in proportion-based meta-analyses that encompass broad regional and temporal variation. Hence, part of the observed asymmetry may reflect structural heterogeneity rather than purely selective publication. Considering these factors, pooled estimates for primary outcomes should be interpreted with caution. We therefore reported random-effects models, I² statistics, and 95% prediction intervals to better account for between-study variance, and we downgraded the certainty of evidence due to potential publication bias and inconsistency.

This study’s strengths include its comprehensive scope, rigorous methodology, and the integration of epidemiological, clinical, psychological, and economic data. However, this systematic review has several limitations. First, although we searched both PubMed and Embase, two comprehensive and widely used databases for clinical medicine, the search strategy could not fully exhaust all potentially relevant studies, and some eligible evidence may still have been missed. Second, as the GBD database does not provide a specific disability weight for WD, we mapped its hepatic and neurologic manifestations to corresponding GBD health states. While this proxy approach follows standard GBD methodology and is widely used for rare diseases, it inevitably introduces uncertainty in burden estimation. Future work using disease-specific disability weights may provide more precise estimates. Third, funnel-plot asymmetry and significant Egger’s test results for several primary outcomes suggest the presence of potential small-study or publication bias. Although such asymmetry is common in rare disease meta-analyses often reflecting heterogeneous study designs, treatment eras, and selective reporting, this possibility should be acknowledged when interpreting the pooled estimates. Fourth, although a comprehensive search strategy was conducted, some relevant synonyms for key outcomes were not explicitly included, which may have resulted in the omission of studies. Fifth, Despite synthesizing data from a large number of studies, the geographic distribution of available evidence remains highly uneven. Estimates for several regions, particularly Africa and Latin America, are based on very limited datasets or isolated single-center cohorts. As a result, the pooled epidemiological and clinical estimates for these under-represented regions carry substantial uncertainty and should be interpreted with caution. The apparent regional differences observed in this review might therefore reflect gaps in data availability rather than true epidemiological variation. Additional high-quality, population-based studies are urgently needed to more accurately characterize the burden of Wilson disease in these areas.

## Conclusion

This systematic review provides a comprehensive and data-driven synthesis of the global burden of Wilson disease across 32 countries. Several novel insights emerged. First, we identified multiple high-prevalence geographic clusters, including Greece (66.7 per 100,000) and regional hotspots in Sardinia, Anhui (China), the Canary Islands, Bahrain, and Israel. Forecasting analyses further suggest continued increases in prevalence in several countries through 2030. Second, major clinical outcomes, including ALF (13.5%), mortality (14%), liver transplantation (13%), and post-transplant mortality (31%). Finally, DALY estimates highlight that national disease burden is highly concentrated, with the United States, China, France, Poland, and South Korea accounting for a disproportionate share of global morbidity and mortality.

Together, these findings integrate epidemiological, clinical, and disease-burden dimensions into a unified global picture of WD, providing an evidence base for early detection, equitable resource allocation, and future research priorities.

## Supplementary information

Below is the link to the electronic supplementary material.

Supplementary Material 1

Supplementary Material 2

Supplementary Material 3

## Data Availability

The datasets used and/or analysed during the current study are available from the corresponding author on reasonable request.
