# Supplementary material for: Global burden of Wilson disease: a comprehensive evidence synthesis
Source: Orphanet J Rare Dis. 2026 Jan 13;21:175. doi: 10.1186/s13023-025-04185-2 (PMC13134261; doi:10.1186/s13023-025-04185-2)
Supplement: Supplementary file 1 — Supplementary Material 1 [file 13023_2025_4185_MOESM1_ESM.docx]

**Supplementary materials**

**Literature Search strategy**

Pubmed

("Wilson's disease" [Mesh] OR "Wilson's disease" [tiab] OR "WD" [tiab] OR "Hepatolenticular Degeneration" [tiab]) AND ("prevalence" OR "incidence" OR "mortality" OR "disease burden" OR "DALY" OR "disability-adjusted life year" OR "quality of life" OR "economic burden" OR "disease cost") NOT (review [publication type] OR case reports [publication type] )

Embase

('wilson disease'/exp OR 'wilson disease':ti,ab OR 'hepatolenticular degeneration':ti,ab OR 'copper metabolism disorder':ti,ab) AND ('prevalence'/exp OR 'incidence'/exp OR 'mortality'/exp OR 'epidemiology'/exp OR 'disease burden':ti,ab OR 'disability adjusted life year':ti,ab OR 'daly':ti,ab OR 'quality of life'/exp OR 'economic burden':ti,ab OR 'cost of illness'/exp) AND 'human'/exp NOT ('review'/it OR 'case report'/it OR 'conference abstract'/it OR ('animal'/exp NOT 'human'/exp)) AND [embase]/lim AND [<2025]/py

**Admission criteria and process**

Only original articles published in English were included. After removing duplicates, all titles and abstracts were screened independently by two reviewers (ST, WH), followed by full-text assessments to determine final eligibility. Studies were included if they reported at least one of the following indicators related to WD: prevalence, incidence of ALF) mortality, quality of life (QOL), or economic burden.

Studies meeting any of the following criteria were excluded: 1) Narrative reviews, systematic reviews, meta-analyses, conference abstracts or case reports; 2) Animal experiments or in vitro studies; 3) Conference abstracts without full text available; 4) Studies lacking original data (e.g., expert opinions, editorials, commentaries); 5) Studies in which WD-related outcomes could not be clearly extracted or were not separately reported; 6) Studies with fewer than 4 WD patients.

Discrepancies in study selection were resolved through discussion or by consultation with a third reviewer.

**Calculation of Disability-Adjusted Life Years (DALYs)**

DALYs were calculated according to the Global Burden of Disease (GBD) study framework. DALYs represent the sum of years of life lost (YLL) due to premature mortality and years lived with disability (YLD). YLL was computed as the number of deaths multiplied by the standard life expectancy at the age of death (YLL = Σ[Deathsₐ × LEₐ]), where LEₐ was based on the WHO reference life table (86 years at birth). YLD was estimated using the prevalence-based approach: YLD = Prevalence × Disability Weight (DW).

**Estimation of DW**

Because WD-specific weights are not available in the GBD dataset, analogous health states were selected to represent the major hepatic and neurologic manifestations of WD. DW were obtained from the Institute for Health Metrics and Evaluation (IHME) GBD 2019 and 2021 DW catalogs (Supplementary Table1). Epidemiological data from pooled cohorts indicate that approximately 70% of WD cases present with predominant hepatic manifestations, while 30% show neurologic or psychiatric features. Among hepatic forms, roughly two-thirds are compensated and one-third are decompensated or fulminant at presentation. Within the neurologic/psychiatric spectrum, mild-to-moderate motor impairment, depression, and anxiety are the most common manifestations.
To derive a representative DW for overall WD, a weighted composite was calculated based on the relative frequency of hepatic and neuropsychiatric forms using the formula:

DW_overall_ = (0.70 × DW_hepatic_) + (0.30 × DW_neuropsychiatric_)

where

DW_hepatic_ = (2/3 × 0.178) + (1/3 × 0.540) = 0.299
DW_neuropsychiatric_ = (0.054 + 0.159 + 0.133 + 0.046) / 4 = 0.098

Thus,

DW_overall_ = (0.70 × 0.299) + (0.30 × 0.098) ≈ 0.23

Because DALYs were estimated on an annual prevalence basis rather than lifetime burden, and to maintain comparability with prior GBD studies that standardize moderate chronic conditions near 0.05 (e.g., chronic hepatitis, 0.051; mild neurological disorder, 0.048), the base-case proxy DW for WD was conservatively set at 0.05. This value lies within the interquartile range of mild-to-moderate chronic disease states in the GBD 2019 catalog and is consistent with previous liver disease burden modeling frameworks.

**Statistical Software and Packages**

All statistical analyses were conducted using R software (version 4.3.0). The following R packages were used, with their corresponding CRAN references provided for reproducibility:

1) metafor (Viechtbauer W, 2010; metafor: Meta-Analysis Package for R, Version 4.4-0, https://CRAN.R-project.org/package=metafor)

2) meta (Balduzzi S, Rücker G, Schwarzer G, 2019; meta: General Package for Meta-Analysis, Version 6.5-0, https://CRAN.R-project.org/package=meta)

3) dplyr (Wickham H et al., 2023; dplyr: A Grammar of Data Manipulation, Version 1.1.3, https://CRAN.R-project.org/package=dplyr)

4) tidyr (Wickham H et al., 2023; tidyr: Tidy Messy Data, Version 1.3.0, https://CRAN.R-project.org/package=tidyr)

5) readxl (Wickham H & Bryan J, 2023; readxl: Read Excel Files, Version 1.4.3, https://CRAN.R-project.org/package=readxl)

6) ggplot2 (Wickham H et al., 2016; ggplot2: Elegant Graphics for Data Analysis,

Version 3.4.4, https://CRAN.R-project.org/package=ggplot2)

All forest plots, funnel plots, and diagnostic graphs were generated using functions from metafor and meta.
