# Supplementary material for: Global burden of Wilson disease: a comprehensive evidence synthesis
Source: Orphanet J Rare Dis. 2026 Jan 13;21:175. doi: 10.1186/s13023-025-04185-2 (PMC13134261; doi:10.1186/s13023-025-04185-2)
Supplement: Supplementary file 2 — Supplementary Material 2 [file 13023_2025_4185_MOESM2_ESM.docx]

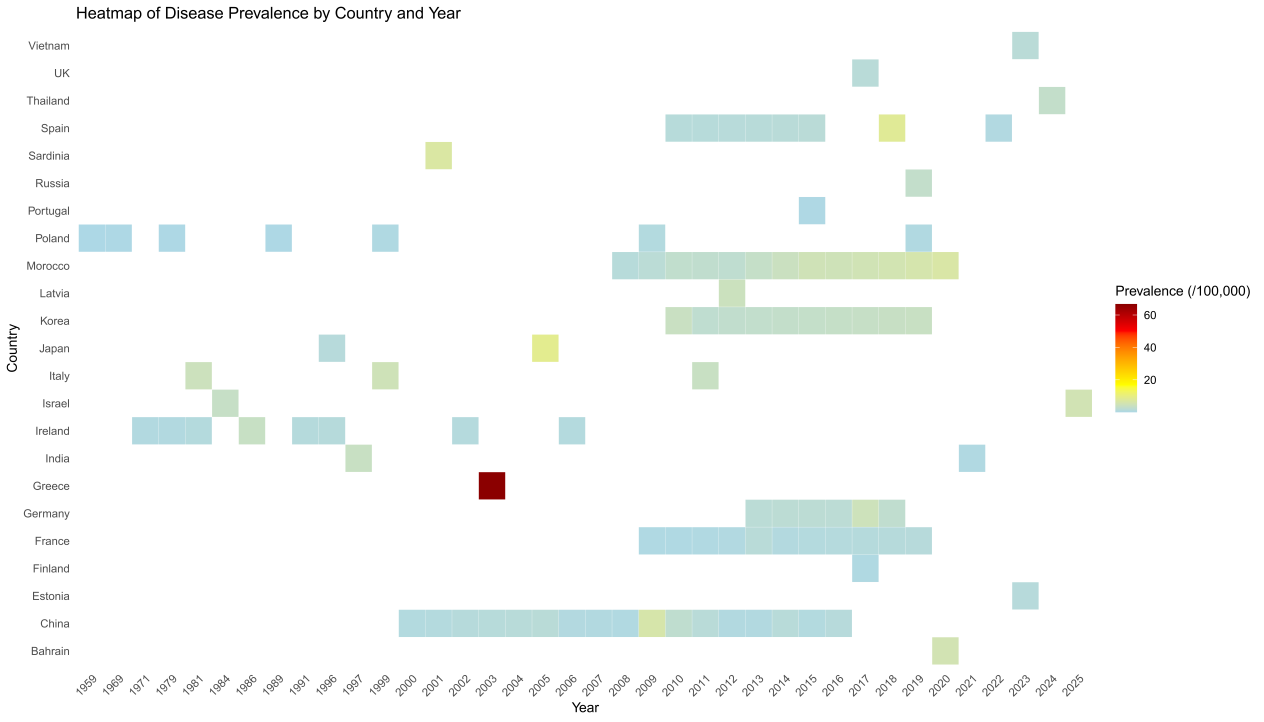


**Supplementary figure1. Heatmap visualization of historical reported prevalence values across countries and years**

Note: Each square represents one reported data point of prevalence. Color intensity corresponds to the reported prevalence per 100,000 population. Data scarcity and reporting heterogeneity are notable across countries and time periods.


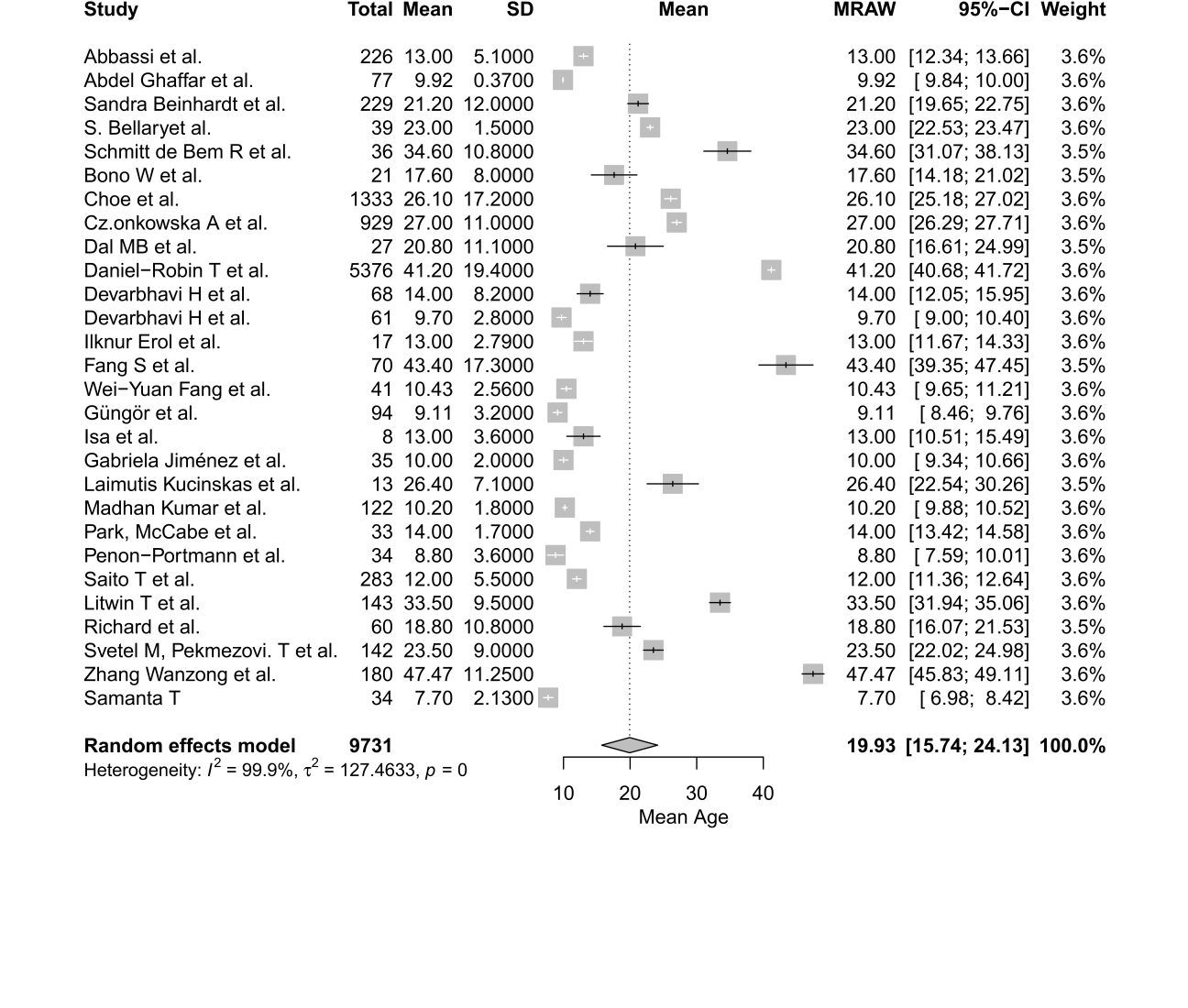


**Supplementary figure2. Forest plot of the pooled mean age of patients with Wilson disease (WD) across included studies.**

MRAW = Mean reported age with weight; CI = Confidence Interval.


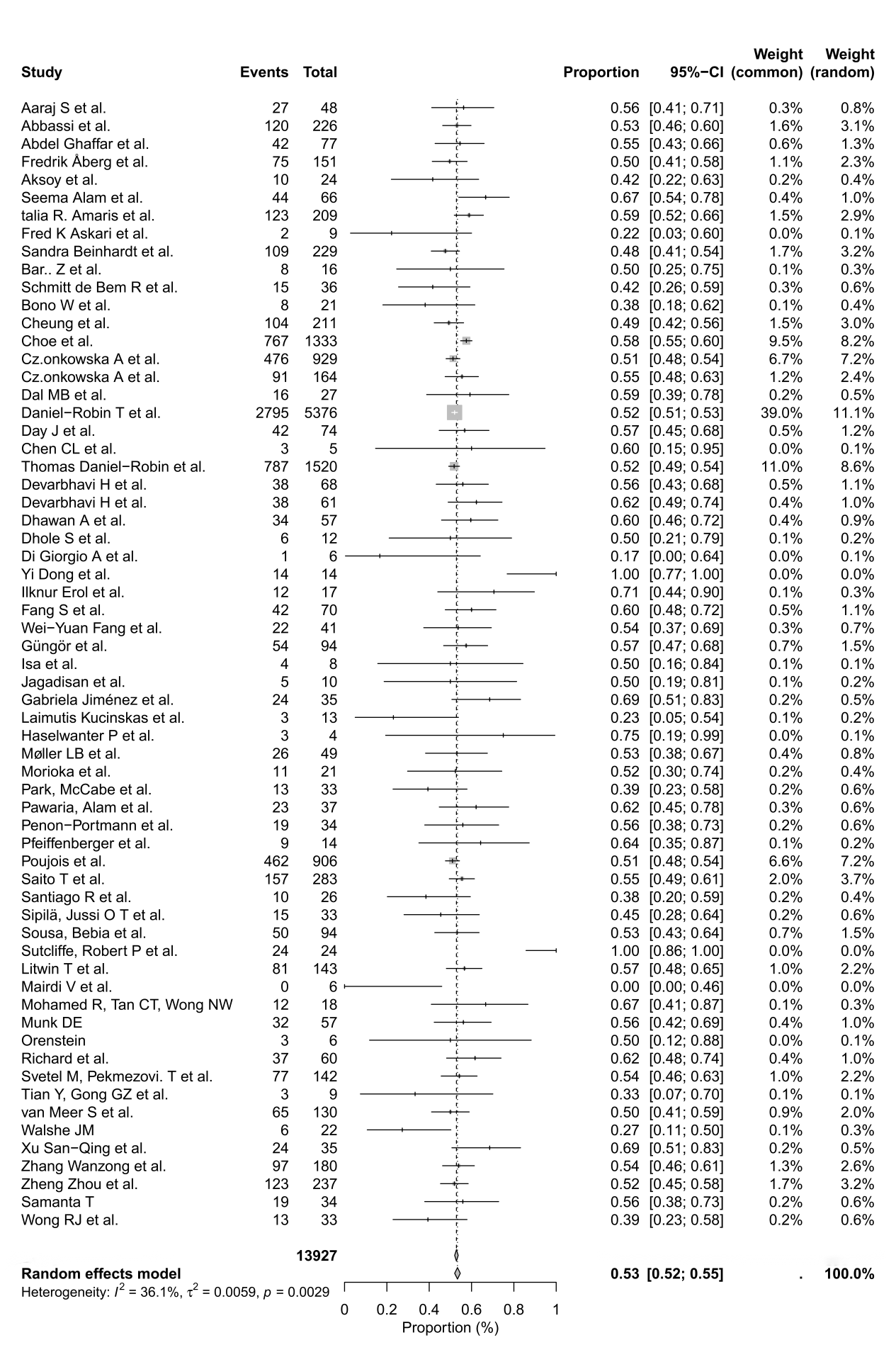


**Supplementary figure3. Forest plot of the pooled proportion of male patients with Wilson disease (WD) across included studies.**

**
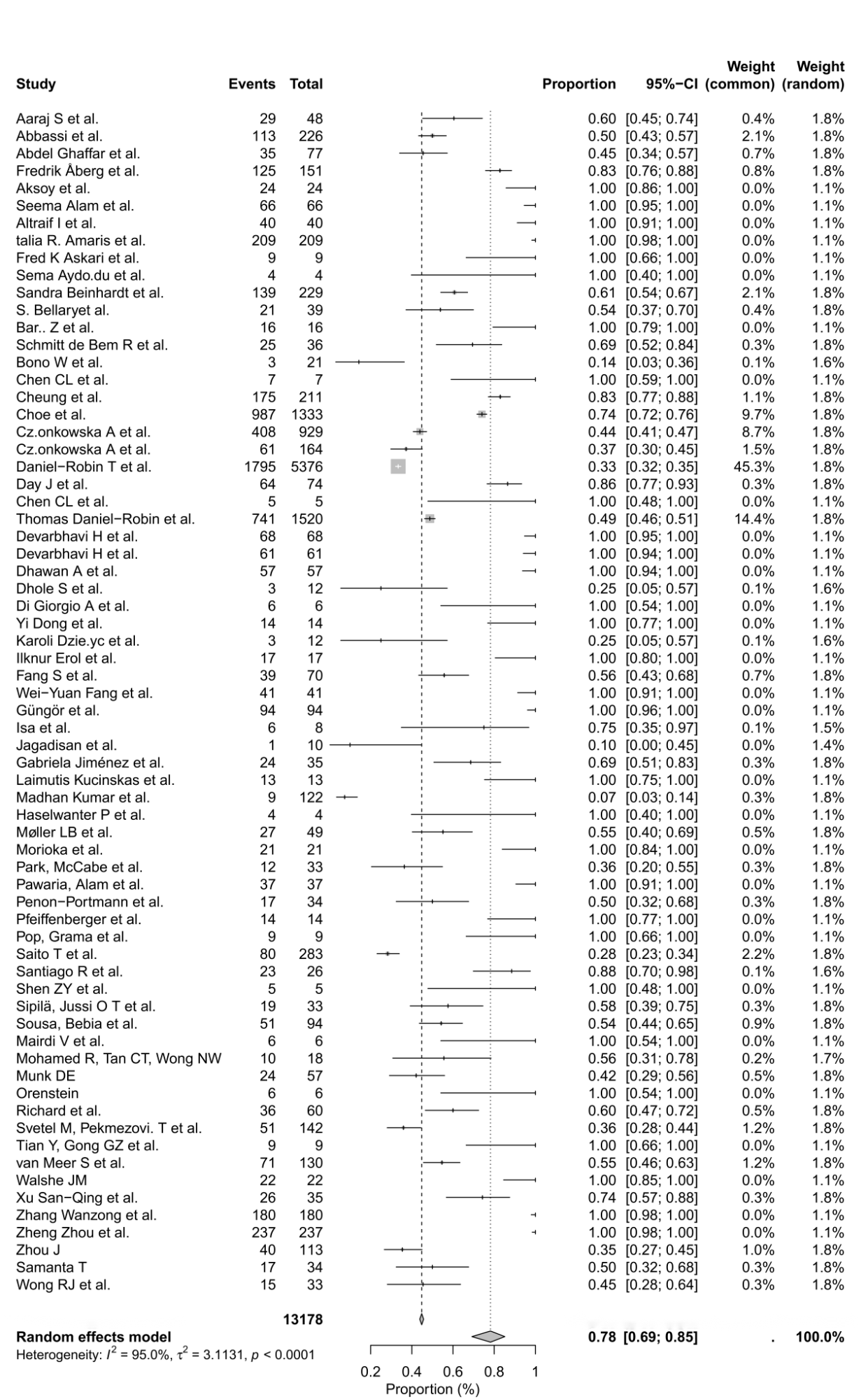
**

**Supplementary figure4. Forest plot of the pooled proportion of Hepatic WD patients across included studies.**

**
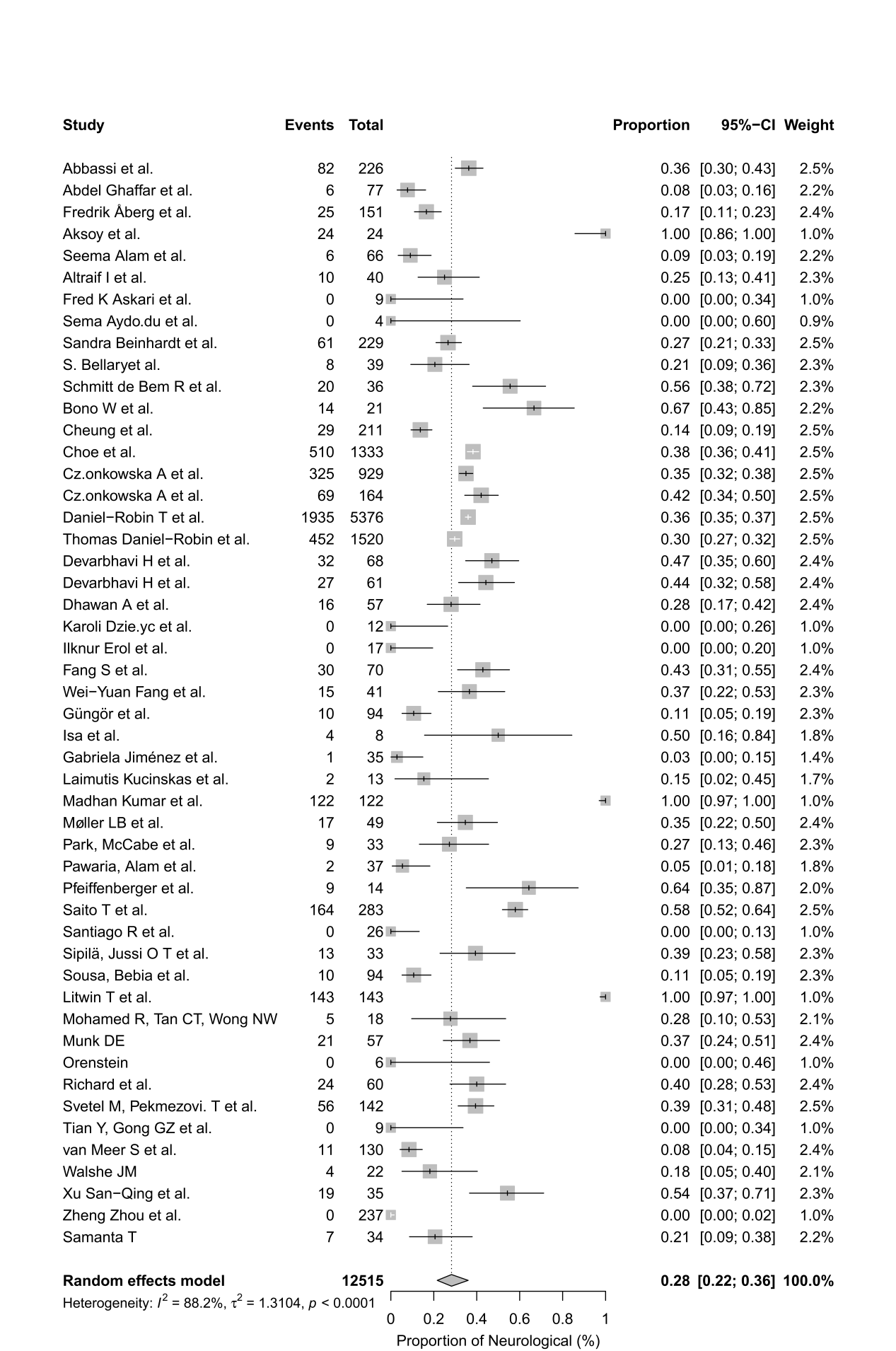
**

**Supplementary figure5. Forest plot of the pooled proportion of Neurological WD patients across included studies.**

**
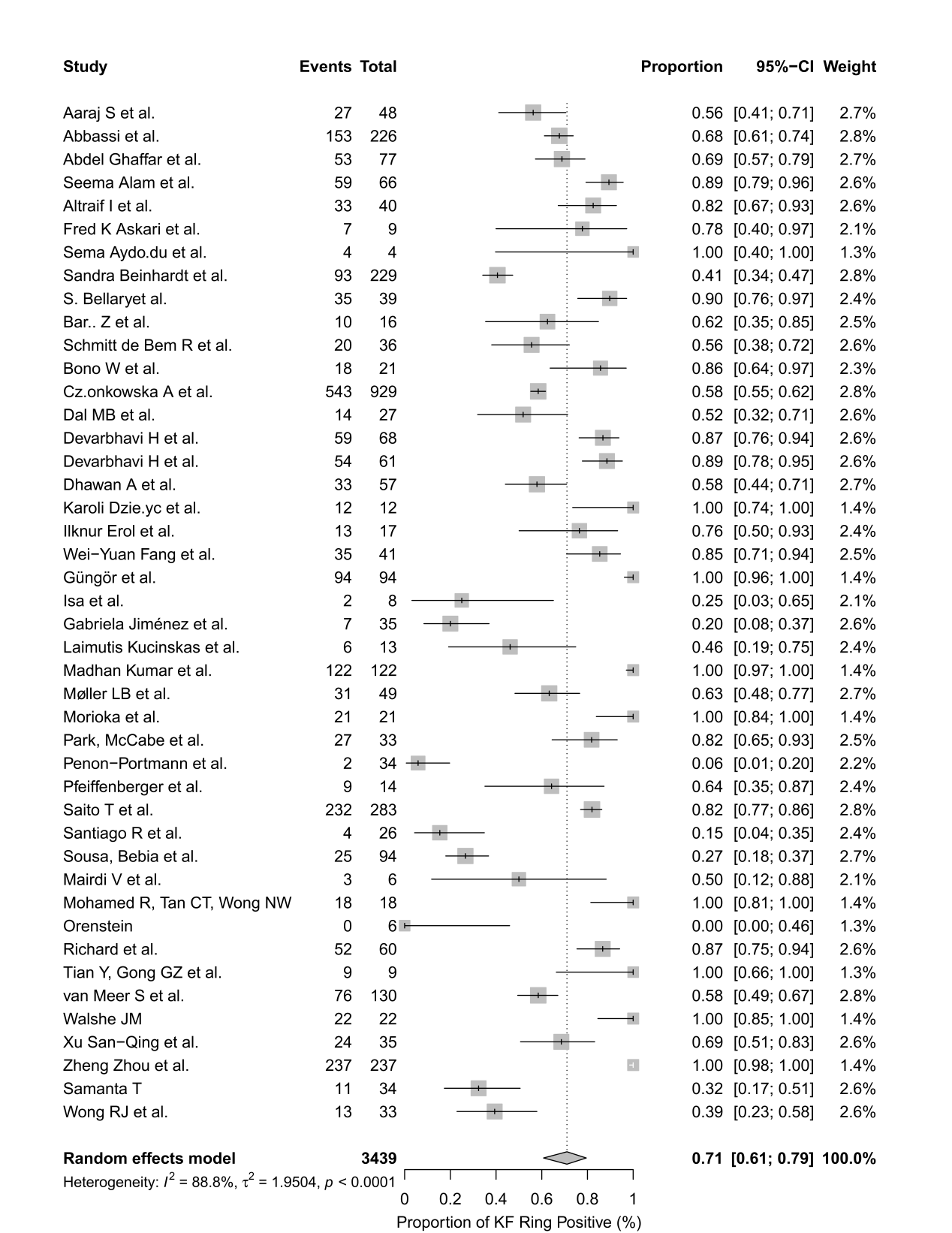
**

**Supplementary figure6. Forest plot of the pooled proportion of KF ring positive WD patients across included studies.**

**
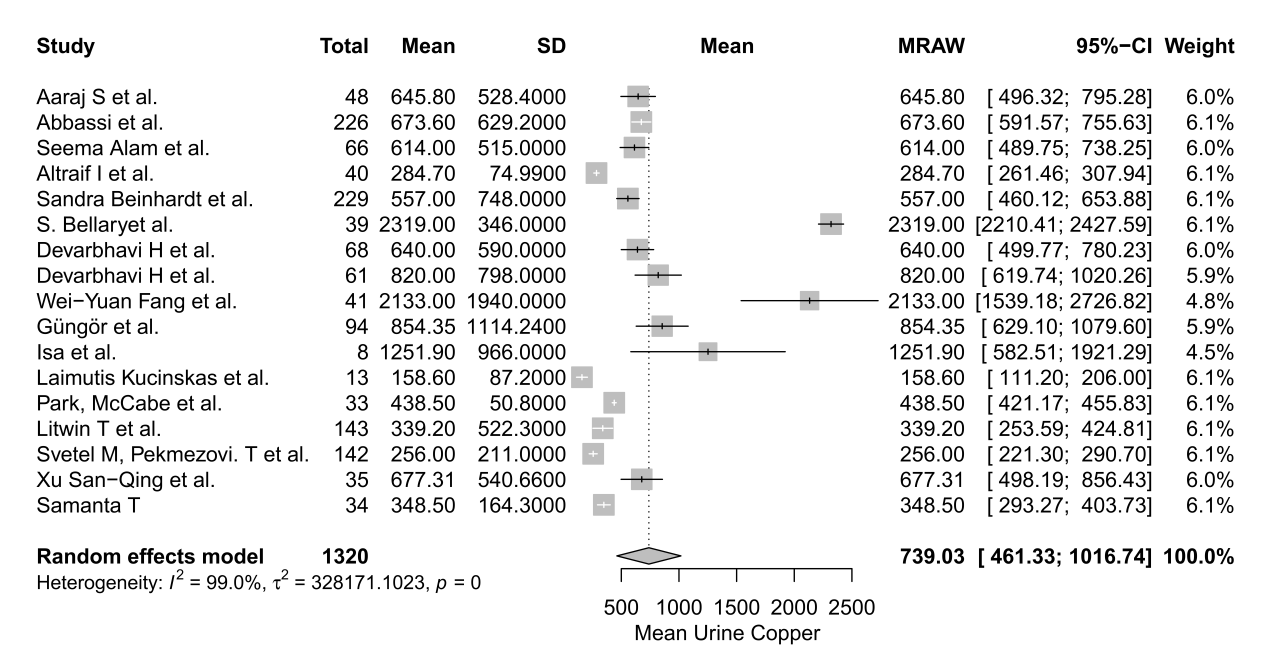
**

**Supplementary figure7. Forest plot of 24-hour urinary copper excretion levels in patients with Wilson disease (WD) across included studies.**

**
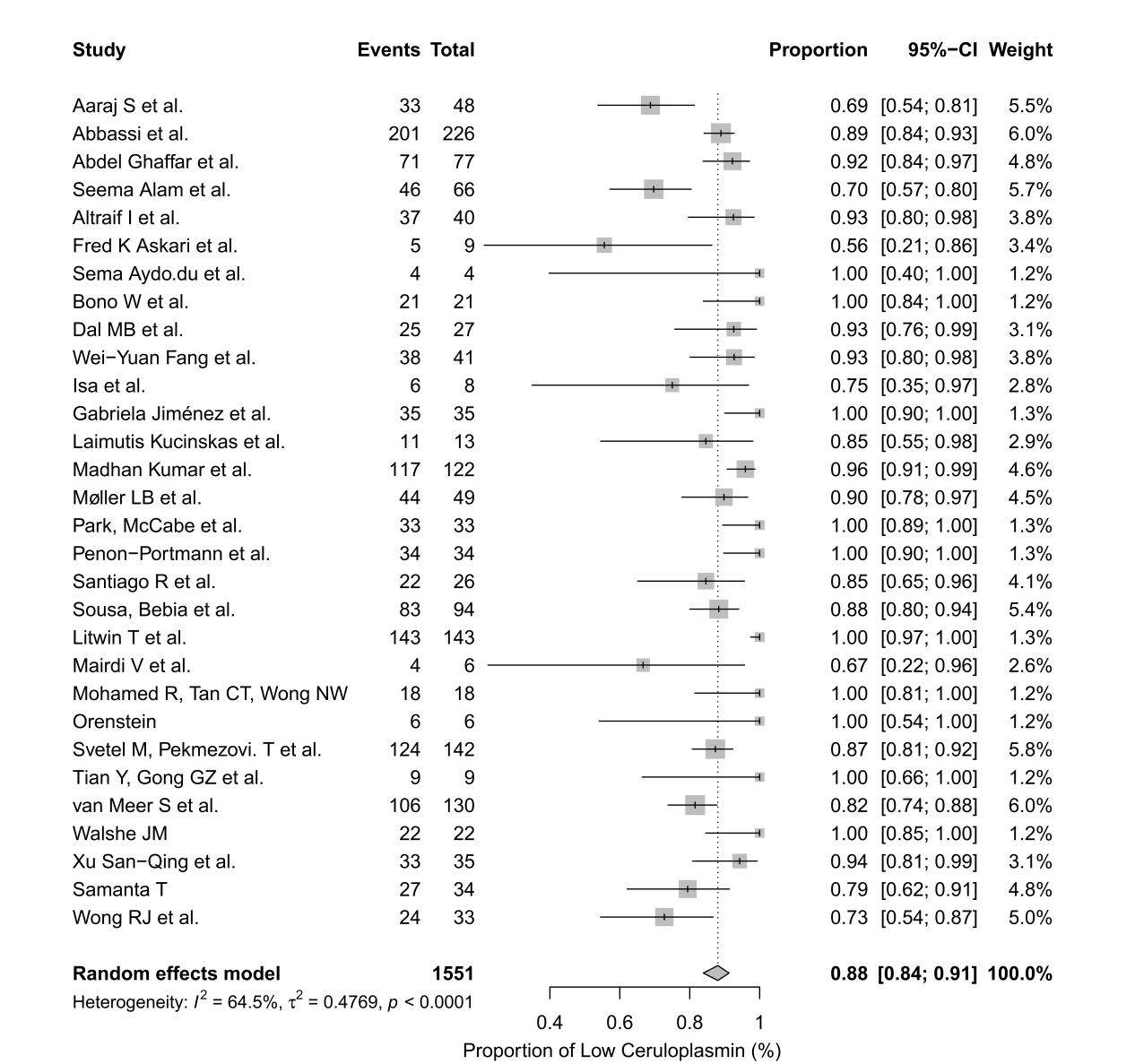
**

**Supplementary figure8. Forest plot of the pooled proportion of Wilson disease (WD) patients with decreased ceruloplasmin levels across included studies.**

**
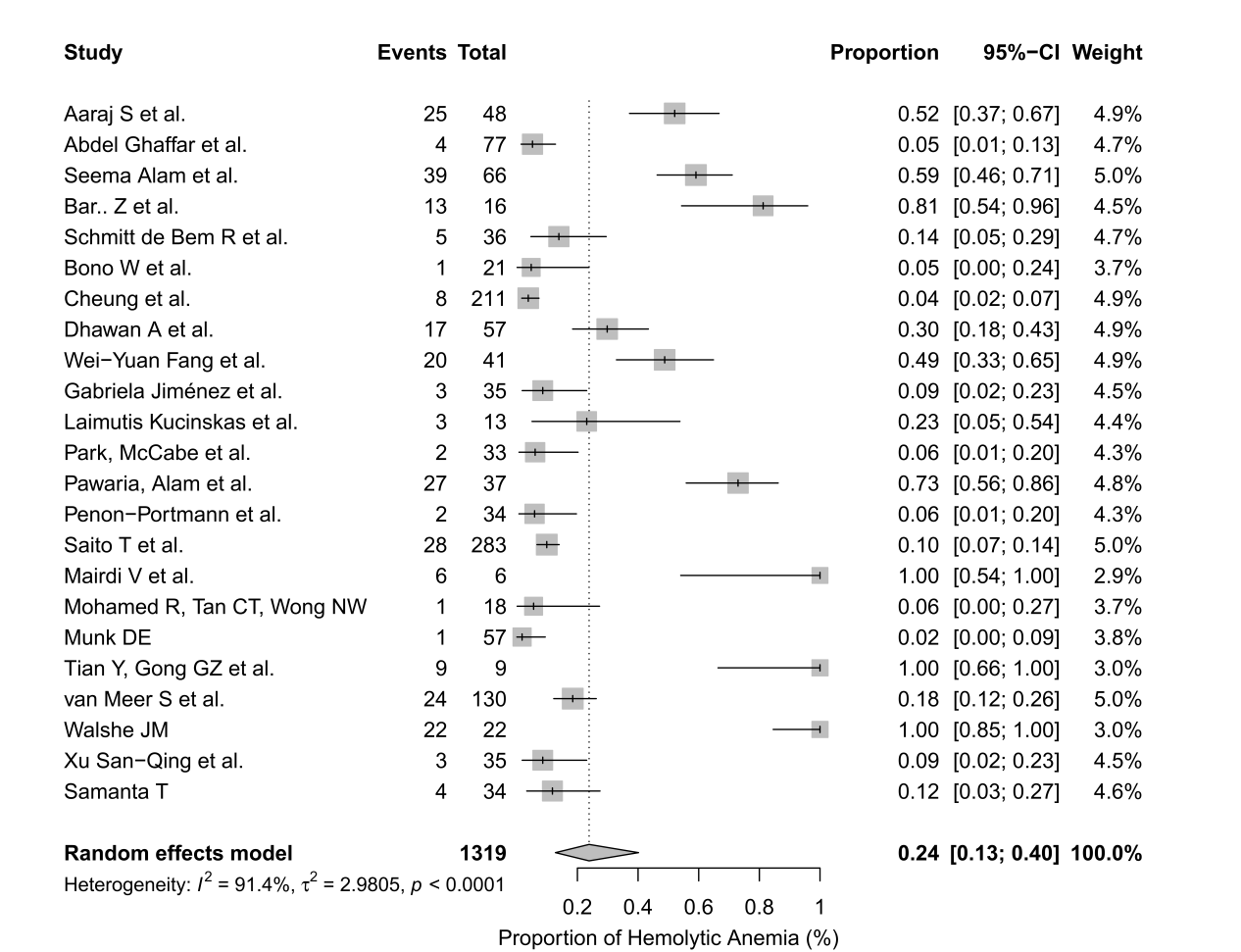
**

**Supplementary figure9. Forest plot of the pooled proportion of Wilson disease (WD) patients with Coombs-negative hemolytic anemia across included studies.**


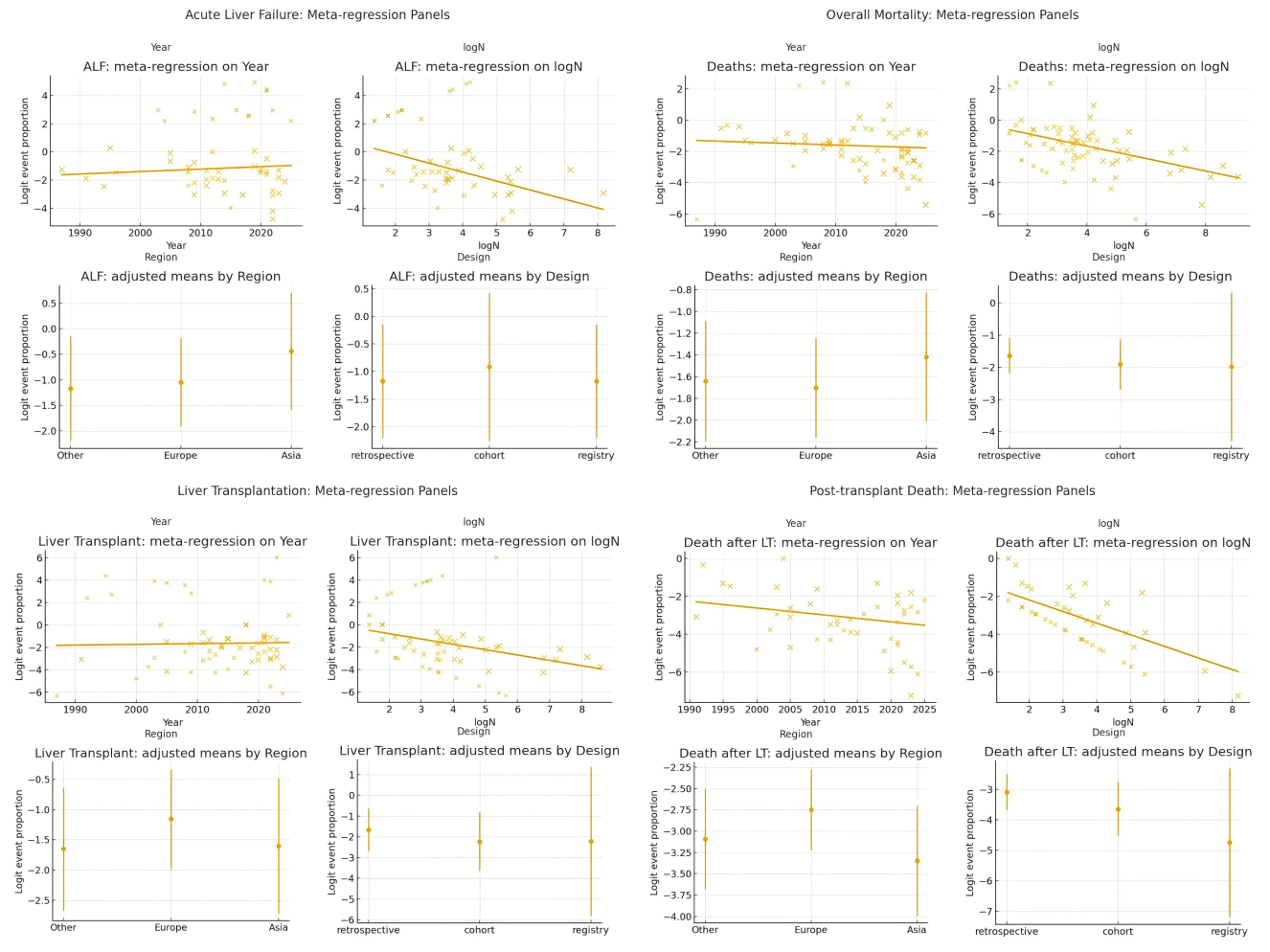


**D**

**C**

**Supplementary figure10. Meta-regression analyses for heterogeneity across primary outcomes.**

Panels show mixed-effects meta-regression plots assessing potential moderators for (A) acute liver failure (ALF), (B) overall mortality, (C) liver transplantation, and (D) post-transplant death.

Note: For each outcome, event proportions were logit-transformed, and regression lines represent fitted relationships with publication year and log-transformed sample size (logN). Error bars in categorical subplots (region and study design) indicate 95% confidence intervals for adjusted means. Notably, smaller studies (lower logN) reported higher event proportions, suggesting small-study effects, while later publication years were associated with decreased mortality following liver transplantation.

**
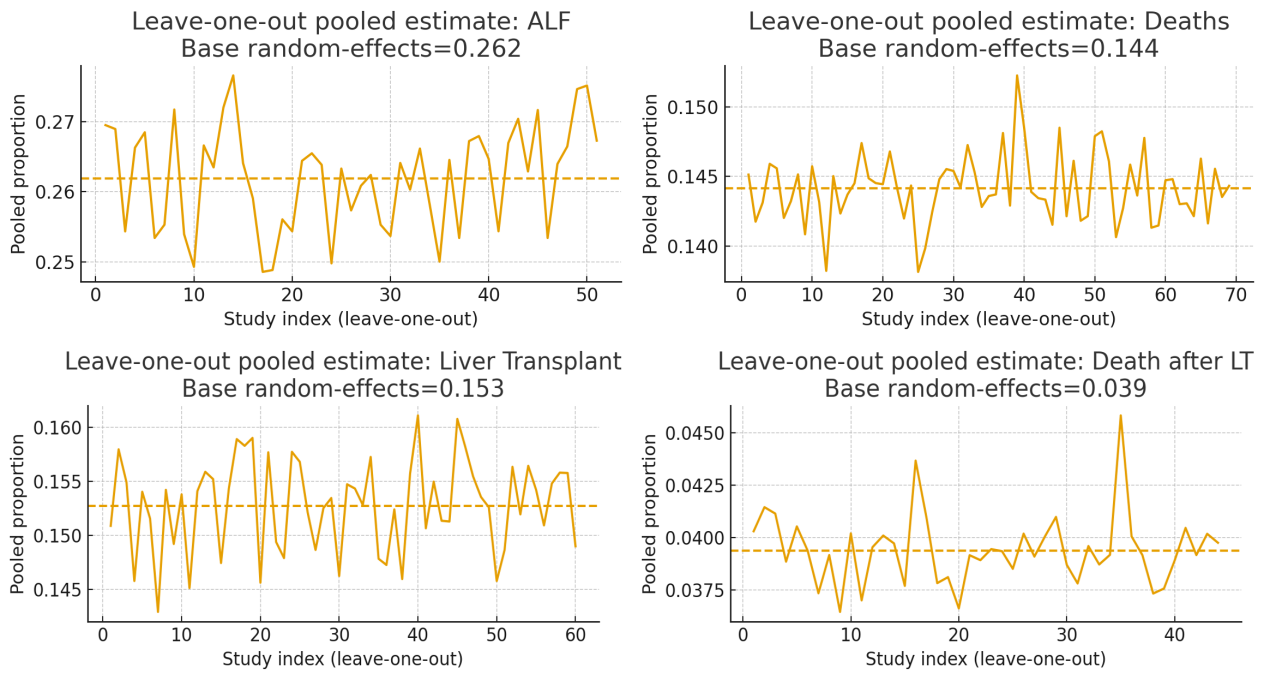
**

**Supplementary figure11. Leave-one-out (LOO) sensitivity analyses for pooled outcomes**

Note: Each panel shows the influence of individual studies on the overall pooled estimates under the random-effects model. The dashed horizontal line indicates the base random-effects proportion for each outcome. The stability of pooled estimates across iterations indicates that no single study exerted an excessive influence on the overall results.

**
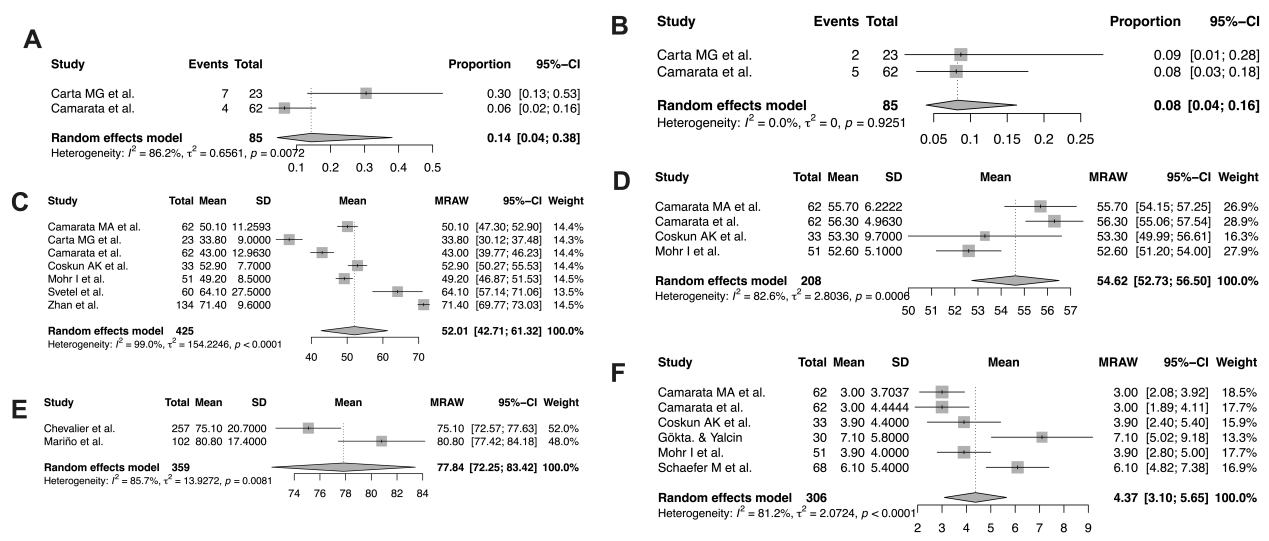
**

**Supplementary figure12. Forest plots summarizing the pooled estimates of neuropsychiatric symptoms, quality of life, and related outcomes in patients with WD across included studies.**

(A) Prevalence of bipolar disorder. (B) Prevalence of panic disorder. (C) Pooled SF-12 mental component summary (MCS) scores. (D) Pooled SF-12 physical component summary (PCS) scores. (E) Pooled EQ-VAS scores. (F) Pooled PHQ-9 scores.

Note: Pooled proportions and 95% confidence intervals (CIs) were estimated using random-effects models for prevalence outcomes (A–H), and pooled means with 95% CIs were estimated for continuous variables (I–L). The weight of each study is indicated. Heterogeneity statistics (I², χ², and p-value) are provided for each analysis.

**A**

**B**

**
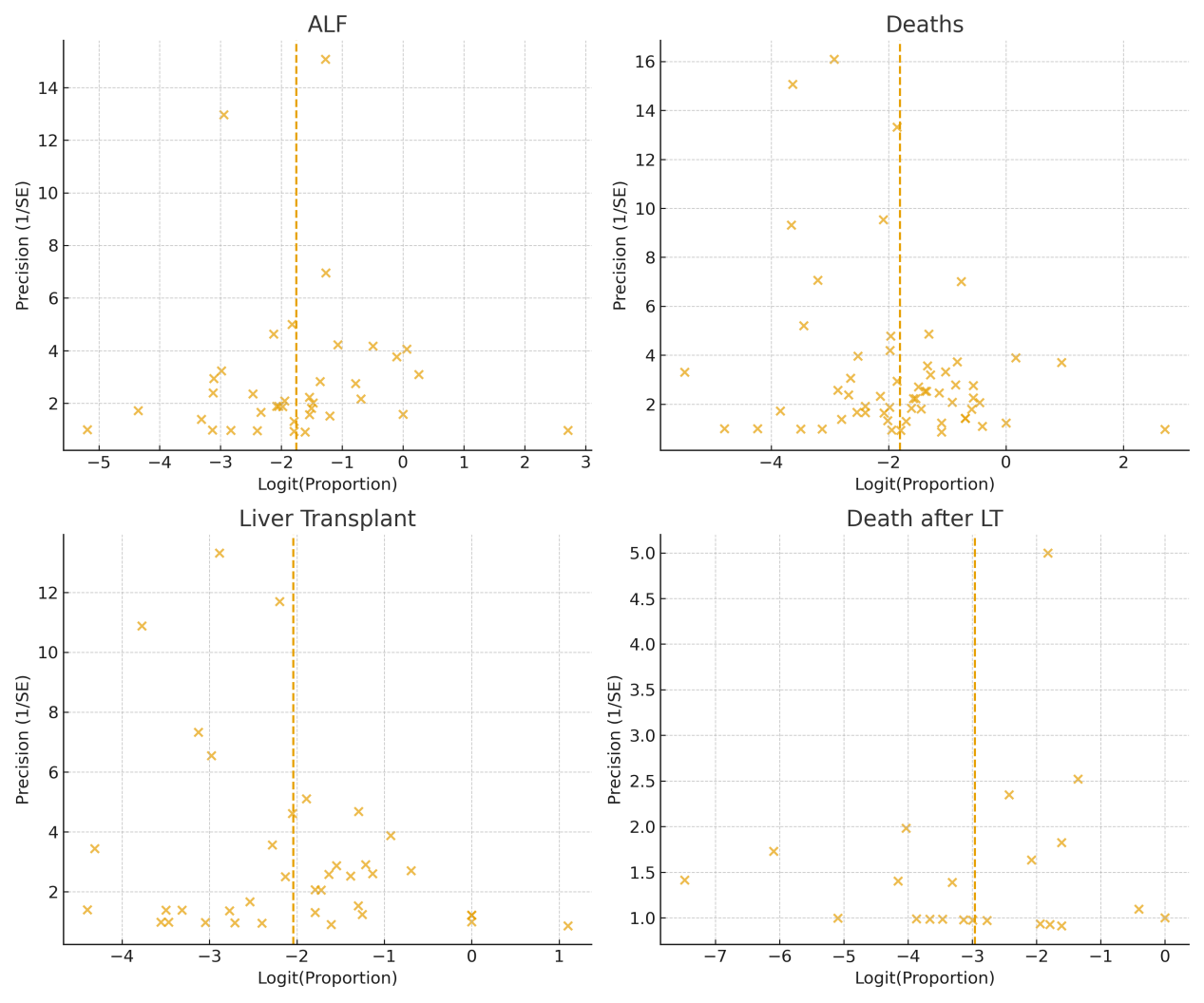
Supplementary figure13. Funnel plots for clinical outcomes (acute liver failure, deaths, liver transplantation, and post-transplant death).**

**
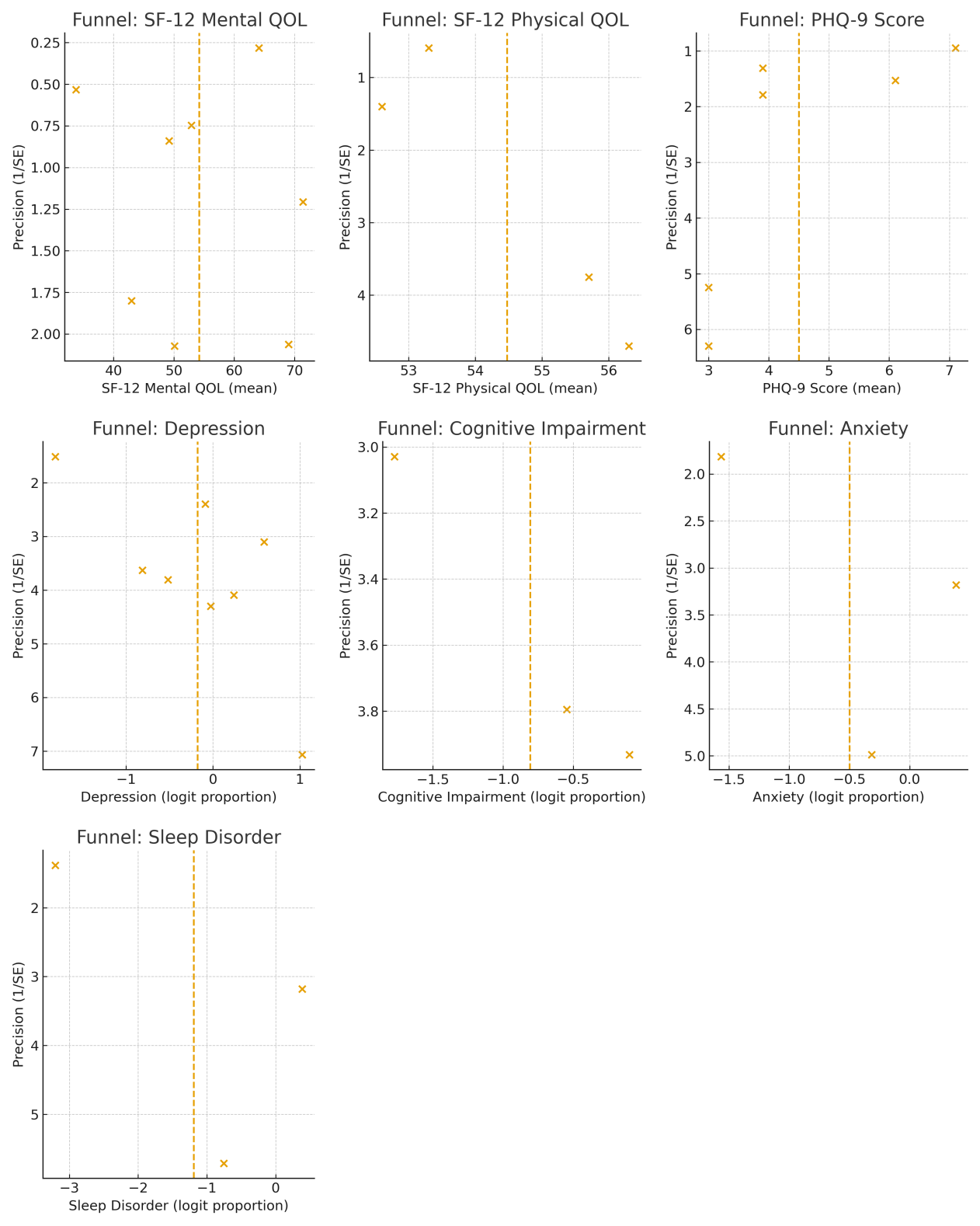
**

**Supplementary figure14. Funnel plots for quality-of-life outcomes (SF-12 Mental and Physical QOL, PHQ-9, depression, anxiety, cognitive impairment, and sleep disorder).**
