# Supplementary material for: Global burden of Wilson disease: a comprehensive evidence synthesis
Source: Orphanet J Rare Dis. 2026 Jan 13;21:175. doi: 10.1186/s13023-025-04185-2 (PMC13134261; doi:10.1186/s13023-025-04185-2)
Supplement: Supplementary file 3 — Supplementary Material 3 [file 13023_2025_4185_MOESM3_ESM.docx]

## **Supplementary Table 1. Proxy disability weights (DWs) applied for WD states**

| **WD Clinical Manifestation** | **Corresponding GBD Health State** | **GBD Code (2019 catalog)** | **Disability Weight (Mean [95% UI])** |
| --- | --- | --- | --- |
| **Compensated hepatic form** | Cirrhosis, compensated | B.4.2.1 | 0.178 (0.118–0.253) |
| **Decompensated hepatic form** | Cirrhosis, decompensated (with ascites / encephalopathy / bleeding) | B.4.2.2 | 0.540 (0.377–0.686) |
| **Acute hepatic failure / fulminant WD** | Acute hepatitis, severe | B.4.1.2 | 0.210 (0.138–0.294) |
| **Neurological WD (motor symptoms)** | Motor impairment, moderate / Parkinsonism | N.2.3 | 0.054 (0.037–0.076) |
| **Neuropsychiatric WD – Depression** | Depressive disorder, moderate | M.3.2 | 0.159 (0.106–0.225) |
| **Neuropsychiatric WD – Anxiety** | Anxiety disorder, moderate | M.3.3 | 0.133 (0.088–0.190) |
| **Residual cognitive / functional impairment** | Cognitive impairment, mild | N.2.1 | 0.046 (0.030–0.066) |
| **Overall mean proxy used for base-case DALY estimate** | Weighted average of above | — | **≈ 0.05** |

**Supplementary Table 2: Overview of Included Epidemiological Studies**

| **No.** | **Author** | **PMID** | **Publication Year** | **Region** | **Annual Incidence Rate (/100,000)** | **Allele Frequency (%)** | **Carrier Frequency** | **Family History (%)** | **Study Period** | **Total Cases** | **Data Source** | **Year** | **Male Population（×10⁴）** | **Female Population（×10⁴）** | **Total Population（×10⁴）** | **Male WD Cases** | **Female WD Cases** | **Total WD Cases** | **Male Prevalence (/100,000)** | **Female Prevalence (/100,000)** | **Total Prevalence (/100,000)** | **Notes** |
| --- | --- | --- | --- | --- | --- | --- | --- | --- | --- | --- | --- | --- | --- | --- | --- | --- | --- | --- | --- | --- | --- | --- |
| 1 | Abbassi et al. | 38588792 | 2024 | Morocco | 3.88 | 0.15 | – | – | 2008–2020 | 226 | 5 university hospital centers | 2008 | 19.74 | 17.57 | 37.31 | 2 | 3 | 5 | 1.01 | 1.71 | 1.34 |  |
|  |  |  |  |  |  |  |  |  |  |  |  | 2009 | 20.26 | 18.97 | 39.23 | 3 | 4 | 7 | 1.48 | 2.11 | 1.78 |  |
|  |  |  |  |  |  |  |  |  |  |  |  | 2010 | 20.75 | 20.68 | 41.43 | 4 | 7 | 11 | 1.93 | 3.38 | 2.66 |  |
|  |  |  |  |  |  |  |  |  |  |  |  | 2011 | 21.25 | 22.6 | 43.85 | 6 | 5 | 11 | 2.82 | 2.21 | 2.51 |  |
|  |  |  |  |  |  |  |  |  |  |  |  | 2012 | 21.71 | 24.29 | 46 | 7 | 4 | 11 | 3.22 | 1.65 | 2.39 |  |
|  |  |  |  |  |  |  |  |  |  |  |  | 2013 | 22.07 | 25.32 | 47.38 | 11 | 4 | 15 | 4.99 | 1.58 | 3.17 |  |
|  |  |  |  |  |  |  |  |  |  |  |  | 2014 | 22.26 | 25.41 | 47.67 | 9 | 10 | 19 | 4.04 | 3.94 | 3.99 |  |
|  |  |  |  |  |  |  |  |  |  |  |  | 2015 | 22.32 | 24.81 | 47.13 | 12 | 11 | 23 | 5.38 | 4.43 | 4.88 |  |
|  |  |  |  |  |  |  |  |  |  |  |  | 2016 | 22.32 | 23.94 | 46.27 | 14 | 8 | 22 | 6.27 | 3.34 | 4.76 |  |
|  |  |  |  |  |  |  |  |  |  |  |  | 2017 | 22.29 | 23.21 | 45.5 | 9 | 14 | 23 | 4.04 | 6.03 | 5.06 |  |
|  |  |  |  |  |  |  |  |  |  |  |  | 2018 | 22.14 | 22.64 | 44.78 | 10 | 14 | 24 | 4.52 | 6.18 | 5.36 |  |
|  |  |  |  |  |  |  |  |  |  |  |  | 2019 | 21.87 | 22.4 | 44.27 | 10 | 16 | 26 | 4.57 | 7.14 | 5.87 |  |
|  |  |  |  |  |  |  |  |  |  |  |  | 2020 | 21.5 | 22.38 | 43.88 | 8 | 21 | 29 | 3.72 | 9.38 | 6.61 |  |
| 2 | Pablo Alonso—Castellano et al. | 39322449 | 2025 | Spain | – | p.Met645Arg: 0.7%; p.Leu708Pro: 2 ; p.His1069Gln: 0.08; p.Gly869Arg: 1.1 | – | 33 | 1970–2021 | 320 | AEEH WD Registry, Spain | 2022 | ~2000 | ~2000 | 4000 | 141 | 119 | 260 | 0.71 | 0.6 | 0.65 |  |
| 3 | T.P. Almdal et al. | 2010159 | 1991 | Denmark | – | – | – | – | 1981–1985 | 6 | Danish national Inpatient Registry | 1985 | ~260 | ~270 | ~512.5 | 2 | 4 | 6 | 0.15 | 0.3 | 0.24 | Mild cases not hospitalized may be missed |
| 4 | Yury A Barbitoff et al. | 31482689 | 2019 | Russia | – | 0.13 | – | – | 2019 | 4 | 694 WES samples, Northwestern Russia | 2019 | – | – | – | 0 | 4 | 4 | – | – | 2.9 | No confirmed WD cases, carrier rate only |
| 5 | Bonné—Tamir B et al. | 2382969 | 1990 | Israel | – | – | – | – | 1958–1989 | 55 | Populationnabased, 1984 population used | 1984 | – | – | – | – | – | – | – | – | Druze: 3.33；Yemenite Jews: 1.11；Iranian Jews: 2.50；Arabs: 1.00；Ashkezi Jews: 0.25 | incidence calculated based on 1984 data |
| 6 | Bylstra Y et al. | 29961769 | 2018 | Singapore | – | 1.03 | – | – | – | – | Singapore Exome Consortium | – | – | – | 0.48 | – | – | – | – | – | – | Based on ATP7B pathogenic variant carrier frequency 0.97%; no actual prevalence reported |
| 7 | Cheng et al. | 24718822 | 2014 | China(Anhui) | – | 0.56 (estimated via HWE) | – | 66.7 (6/9) | until 2009 | 9 | Mass screening in 3 counties of Anhui Province | – | 7.21 | 8.12 | 15.33 | 4 | 5 | 9 | 5.55 | 6.15 | 5.87 | Population—based cross sectional survey; allele frequency estimated assuming Hardy—Weinberg equilibrium |
| 8 | Cheung et al. | 29209112 | 2017 | China, Hong Kong | 0.144 | – | – | 11.9 | 2000–2016 | 211 | CDARS database + Queen Mary Hospital registry | 2000 | 32.8 | 33.9 | 66.7 | 4 | 7 | 11 | 1.22 | 2.06 | 1.65 |  |
|  |  |  |  |  |  |  |  |  |  |  |  | 2001 | 32.8 | 34.3 | 67.1 | 6 | 9 | 15 | 1.83 | 2.62 | 2.24 |  |
|  |  |  |  |  |  |  |  |  |  |  |  | 2002 | 32.8 | 35.6 | 67.4 | 3 | 8 | 11 | 0.91 | 2.25 | 1.63 |  |
|  |  |  |  |  |  |  |  |  |  |  |  | 2003 | 32.6 | 34.7 | 67.3 | 11 | 7 | 18 | 3.37 | 2.02 | 2.67 |  |
|  |  |  |  |  |  |  |  |  |  |  |  | 2004 | 32.7 | 35.2 | 67.8 | 9 | 4 | 13 | 2.75 | 1.14 | 1.92 |  |
|  |  |  |  |  |  |  |  |  |  |  |  | 2005 | 32.6 | 35.5 | 68.1 | 4 | 5 | 9 | 1.23 | 1.41 | 1.32 |  |
|  |  |  |  |  |  |  |  |  |  |  |  | 2006 | 32.7 | 35.9 | 68.6 | 2 | 3 | 5 | 0.61 | 0.84 | 0.73 |  |
|  |  |  |  |  |  |  |  |  |  |  |  | 2007 | 32.8 | 36.3 | 69.2 | 2 | 3 | 5 | 0.61 | 0.83 | 0.72 |  |
|  |  |  |  |  |  |  |  |  |  |  |  | 2008 | 32.9 | 36.7 | 69.6 | 2 | 2 | 4 | 0.61 | 0.54 | 0.57 |  |
|  |  |  |  |  |  |  |  |  |  |  |  | 2009 | 32.8 | 36.9 | 69.7 | 6 | 10 | 16 | 1.83 | 2.71 | 2.3 |  |
|  |  |  |  |  |  |  |  |  |  |  |  | 2010 | 32.9 | 37.3 | 70.2 | 9 | 8 | 17 | 2.74 | 2.14 | 2.42 |  |
|  |  |  |  |  |  |  |  |  |  |  |  | 2011 | 33 | 37.7 | 70.7 | 8 | 3 | 11 | 2.42 | 0.8 | 1.56 |  |
|  |  |  |  |  |  |  |  |  |  |  |  | 2012 | 33.3 | 38.3 | 71.5 | 3 | 2 | 5 | 0.9 | 0.52 | 0.7 |  |
|  |  |  |  |  |  |  |  |  |  |  |  | 2013 | 33.3 | 38.6 | 71.9 | 2 | 3 | 5 | 0.6 | 0.78 | 0.7 |  |
|  |  |  |  |  |  |  |  |  |  |  |  | 2014 | 33.5 | 39 | 72.4 | 2 | 8 | 10 | 0.6 | 2.05 | 1.38 |  |
|  |  |  |  |  |  |  |  |  |  |  |  | 2015 | 33.7 | 39.4 | 73.1 | 6 | 0 | 6 | 1.78 | 0 | 0.82 |  |
|  |  |  |  |  |  |  |  |  |  |  |  | 2016 | 33.8 | 39.6 | 73.4 | 5 | 4 | 9 | 1.48 | 1.01 | 1.23 |  |
| 9 | Alison J Coffey et al. | 23518715 | 2013 | UK | – | H1069Q: 19；M769V: 6 | – | – | 2001–2012 | 181 | Clinical diagnosis + population screening | – | – | – | – | – | – | 181 | – | – | – | Mutation spectrum only; no specific prevalence/incidence data reported |
| 10 | Corinne Collet et al. | 30097039 | 2018 | France | – | 1.578% (pathogenic or likely pathogenic) | – | – | 2015–2017 | Not given | Populationnabased gene screening (n=697) | – | – | – | – | – | – | – | – | – | – | Allele frequency based on 697 general population samples |
| 11 | Członkowska A et al. | 36427277 | 2022 | Poland | 0.59 | – | – | – | 1950s–2019 | 929 | Polish national Reference Center | 1950–1959 | 1320 | 1410 | 2730 | 18 | 3 | 21 | 0.14 | 0.02 | 0.08 |  |
|  |  |  |  |  |  |  |  |  |  |  |  | 1960–1969 | 1450 | 1560 | 3010 | 36 | 18 | 54 | 0.25 | 0.12 | 0.18 |  |
|  |  |  |  |  |  |  |  |  |  |  |  | 1970–1979 | 1580 | 1700 | 3280 | 39 | 31 | 70 | 0.25 | 0.18 | 0.21 |  |
|  |  |  |  |  |  |  |  |  |  |  |  | 1980–1989 | 1710 | 1830 | 3540 | 42 | 25 | 67 | 0.25 | 0.14 | 0.19 |  |
|  |  |  |  |  |  |  |  |  |  |  |  | 1990–1999 | 1830 | 1950 | 3780 | 86 | 88 | 174 | 0.47 | 0.45 | 0.46 |  |
|  |  |  |  |  |  |  |  |  |  |  |  | 2000–2009 | 1860 | 1980 | 3840 | 147 | 168 | 315 | 0.79 | 0.85 | 0.82 |  |
|  |  |  |  |  |  |  |  |  |  |  |  | 2010–2019 | 1860 | 1980 | 3840 | 109 | 119 | 228 | 0.59 | 0.6 | 0.59 |  |
| 12 | Dedoussis GVZ et al. | 15845031 | 2005 | Greece (in mountain villages) |  | p.Q289X: 5.56 | – | 100 | 1980–2005 | 6 | Epidemiological survey in mountain villages | 1978–2003 | – | – |  | 4 | 2 | 6 | 44.4 | 22.2 | 66.7 | Extremely high local incidence: 6/90 in 25—year birth cohort |
| 13 | Li Shan Chen et al. | 38135707 | 2023 | China, Taiwan | – | 2.5 | – | – | 2018–2022 | 464 | Shin Kong Wu HonaSu Memorial Hospital | – | – | – | – | – | – | – | – | – | – | No population hospital—based |
| 14 | Thomas Daniel—Robin et al. | 35793759 | 2022 | France | 0.08–0.23 | – | – | – | 2009–2019 | 1,520 | SNDS (French national insurance claims database) | 2009 | 2475 | 2525 | 5000 | 98 | 95 | 193 | 0.4 | 0.38 | 0.39 |  |
|  |  |  |  |  |  |  |  |  |  |  |  | 2010 | 2482.5 | 2532.5 | 5015 | 112 | 108 | 220 | 0.45 | 0.43 | 0.44 |  |
|  |  |  |  |  |  |  |  |  |  |  |  | 2011 | 2490 | 2540 | 5030 | 135 | 130 | 265 | 0.54 | 0.51 | 0.53 |  |
|  |  |  |  |  |  |  |  |  |  |  |  | 2012 | 2497.5 | 2547.5 | 5045 | 158 | 152 | 310 | 0.63 | 0.6 | 0.61 |  |
|  |  |  |  |  |  |  |  |  |  |  |  | 2013 | 2505 | 2555 | 5060 | 182 | 175 | 357 | 0.73 | 0.68 | 0.71 |  |
|  |  |  |  |  |  |  |  |  |  |  |  | 2014 | 2512.5 | 2562.5 | 5075 | 205 | 198 | 403 | 0.82 | 0.77 | 0.79 |  |
|  |  |  |  |  |  |  |  |  |  |  |  | 2015 | 2520 | 2570 | 5090 | 230 | 222 | 452 | 0.91 | 0.86 | 0.89 |  |
|  |  |  |  |  |  |  |  |  |  |  |  | 2016 | 2527.5 | 2577.5 | 5105 | 255 | 245 | 500 | 1.01 | 0.95 | 0.98 |  |
|  |  |  |  |  |  |  |  |  |  |  |  | 2017 | 2535 | 2585 | 5120 | 280 | 270 | 550 | 1.1 | 1.04 | 1.07 |  |
|  |  |  |  |  |  |  |  |  |  |  |  | 2018 | 2542.5 | 2592.5 | 5135 | 305 | 295 | 600 | 1.2 | 1.14 | 1.17 |  |
|  |  |  |  |  |  |  |  |  |  |  |  | 2019 | 2550 | 2600 | 5150 | 330 | 320 | 650 | 1.29 | 1.23 | 1.26 |  |
| 15 | Fang S et al. | 39261850 | 2024 | Germany | 0.15 | – | – | – | 2013–2018 | 74 | German WIG2 insurance database (~4.5M people) | 2013 | 4010 | 4130 | 8140 | 33 | 24 | 57 | 1.97 | 1.58 | 1.78 | Covers ~7% of German SHI population |
|  |  |  |  |  |  |  |  |  |  |  |  | 2014 | 4000 | 4120 | 8120 | 37 | 25 | 62 | 2.19 | 1.62 | 1.92 |  |
|  |  |  |  |  |  |  |  |  |  |  |  | 2015 | 3990 | 4110 | 8100 | 40 | 23 | 63 | 2.37 | 1.5 | 1.95 |  |
|  |  |  |  |  |  |  |  |  |  |  |  | 2016 | 3980 | 4100 | 8080 | 35 | 25 | 60 | 2.1 | 1.61 | 1.88 |  |
|  |  |  |  |  |  |  |  |  |  |  |  | 2017 | 3970 | 4090 | 8060 | 40 | 27 | 67 | 2.51 | 1.85 | 2.19 |  |
|  |  |  |  |  |  |  |  |  |  |  |  | 2018 | 3960 | 4080 | 8040 | 41 | 33 | 74 | 2.59 | 2.28 | 2.44 |  |
| 16 | Fang S et al. | 39806721 | 2024 | USA | – | – | – | – | 2016–2019 | 2,115 | Komodo Health insurance database (120M covered) | – | 47520 | 49120 | 96640 | 697 | 784 | 1,481 | 2.12 | 2.11 | 2.12 |  |
| 17 | Yuqin Fang et al. | 37031186 | 2023 | China | — | – | 4.06 | – | 2020 | 320 | Anhui Women and Children Hospital ECS study | 2020 | 0.0084 | 0.0243 | 0.0327 | – | – | 13 carriers | – | – | – | Based on reproductive age ECS screening; not full population prevalence |
| 18 | García—Villarreal et al. | 11093740 | 2000 | Spain (Canary Is.) | 38.5 (Northeast region) | – | – | – | — | 24 (surviving) | Two major hospitals, Gran Canaria Island | 2000 | — | — | 7.38 (NE region) | — | — | 38 | — | — | 38.5 (NE region) |  |
| 19 | García—Villarreal et al. | 33159804 | 2021 | Spain (Canary Is.) | 8.1 (whole island) | Leu708Pro mutation 68.6 | – | — | 2016–2018 | 70 (from 50 families) | Two hospitals, Gran Canaria Island | 2018 | — | — | 86.58 | 41 | 29 | 70 | — | — | 8.09 |  |
| 20 | Giagheddu et al. | 3876678 | 1985 | Italy (Sardinia) | – | – | 48.5% (33/68) | – | 1902–1983 | 68 | Sardinia island medical records | 1981 | 79.29 | 79.29 | 158.59 | 44 | 24 | 68 | 5.55 | 3.03 | 4.29 |  |
| 21 | Gialluisi et al. | 23486543 | 2013 | Italy (Sardinia) | – | 1.91% | – | – | 2011 | 178 | Sardinia WD patients genetic data | 2011 | – |  | 167.24 | – | – | 178 | – | – | 3.66 |  |
| 22 | Isa et al. | 39559689 | 2024 | Bahrain | – | – | – | 75% | 2002–2024 | 8 | Salmaniya Medical Center | 2020 | 74.5 | 75.5 | 150 | 4 | 4 | 8 | 5.37 | 5.3 | 5.33 |  |
| 23 | Eun Sun Jang, Hwa Young Choi et al. | 38565173 | 2024 | Korea | 0.11 | — | – | — | 2010–2020 | 622 | NHIS national insurance database | 2010 | 2467 | 2486 | 4953 | 689 | 493 | 1,840 | 4.34 | 3.26 | 3.8 |  |
|  |  |  |  |  |  |  |  |  |  |  |  | 2011 | 2481 | 2500 | 4981 | 746 | 548 | 1,294 | 2.75 | 2.04 | 2.39 |  |
|  |  |  |  |  |  |  |  |  |  |  |  | 2012 | 2495 | 2513 | 5008 | 800 | 590 | 1,390 | 2.99 | 2.22 | 2.61 |  |
|  |  |  |  |  |  |  |  |  |  |  |  | 2013 | 2508 | 2526 | 5034 | 851 | 623 | 1,474 | 3.23 | 2.37 | 2.8 |  |
|  |  |  |  |  |  |  |  |  |  |  |  | 2014 | 2520 | 2538 | 5058 | 884 | 658 | 1,542 | 3.39 | 2.53 | 2.96 |  |
|  |  |  |  |  |  |  |  |  |  |  |  | 2015 | 2531 | 2549 | 5080 | 921 | 679 | 1,600 | 3.58 | 2.63 | 3.11 |  |
|  |  |  |  |  |  |  |  |  |  |  |  | 2016 | 2541 | 2559 | 5100 | 945 | 714 | 1,659 | 3.71 | 2.8 | 3.25 |  |
|  |  |  |  |  |  |  |  |  |  |  |  | 2017 | 2550 | 2568 | 5118 | 977 | 737 | 1,714 | 3.88 | 2.92 | 3.4 |  |
|  |  |  |  |  |  |  |  |  |  |  |  | 2018 | 2558 | 2576 | 5134 | 1,002 | 759 | 1,761 | 4.04 | 3.03 | 3.54 |  |
|  |  |  |  |  |  |  |  |  |  |  |  | 2019 | 2565 | 2583 | 5148 | 1,028 | 778 | 1,806 | 4.21 | 3.15 | 3.68 |  |
| 24 | Ja—Hyun Jang et al. | 28515472 | 2017 | Korea | – | 1.15 | 0.0722222 | — | 2014–2016 | 14,835 newborn heel screening | Newborn heel blood screening | 2016 | – | – | – | – | – | – | – | – | – |  |
| 25 | Simone—Svea Janka et al. | 33197948 | 2020 | Germany | 0.32 (2017) | — | – | — | 2009–2017 | 3,261 (2017 treated patients) | - | 2017 | – | – | 7280 | – | – | 3,174 | – | – | 4.5–4.6 |  |
| 26 | Gu—Hwan Kim et al. | 18652531 | 2008 | Korea | 0.32 (estimated) | 0.57 (4 mutations) | 0.0113 | — | 2007–2008 | 476 newborns | Newborn heel blood genetic test | 2008 | – | – | – | – | – | – | – | – | – |  |
| 27 | Laimutis Kucinskas et al. | 18855987 | 2008 | Lithuania | — | 46.2 (p.H1069Q) | 68.7% (family carriers) | 100 (familial) | 1999–2008 | 13 patients + 16 relatives | Kaunas Univ Hospital Hepatology | 2008 | – | – | – | 3 | 10 | 13 | – | – | – |  |
| 28 | Wen—bin Hu et al. | 21600116 | 2011 | China (Anhui) | 2.66 | — | — | — | 2009 | 7 | Urban/rural population screening | 2009 | 5.15 | 6.13 | 11.28 | 3 | 4 | 7 | 5.82 | 6.53 | 6.21 |  |
| 29 | Krumi— A et al. | 19062534 | 2008 | Latvia | ≥0.39 (estimated) | 52.5% (H1069Q) | ~1/80 (estimated) | — | 2005–2007 | 40 referred cases (37 families) | Referral cases | — | – | – | – | 21 | 19 | 40 | — | — | 0.39(estimated) | Based on healthy population carrier rate (1/157) and mutation frequency; estimated prevalence ≥1/25,600 (~0.39/100,000) |
| 30 | Kumar M et al. | 39535360 | 2025 | India | – | — | 1/69 | 19.50% | — | 128 multicenter referral cases (iCROWD consortium) | — | 2021 | – | – | 14 | — | — | 128 | — | — | 0.54 (estimated) | estimated based on Hardy—Weinberg equilibrium |
| 31 | Lai CH, Tseng HF | 20100230 | 2010 | China, Taiwan | 0.27 (2001–2005) | — | — | — | 2000–2005 | 495 | China(Taiwan) Health Insurance | 2000 | **1088** | **1139** | **2228** | 83 | 105 | 188 | 0.76 | 0.92 | 0.84 |  |
|  |  |  |  |  |  |  |  |  |  |  |  | 2001 | 1096 | 1144 | 2241 | 94 | 112 | 206 | 0.86 | 0.98 | 0.92 |  |
|  |  |  |  |  |  |  |  |  |  |  |  | 2002 | 1104 | 1149 | 2252 | 121 | 143 | 264 | 1.1 | 1.25 | 1.17 |  |
|  |  |  |  |  |  |  |  |  |  |  |  | 2003 | 1109 | 1152 | 2260 | 134 | 156 | 290 | 1.21 | 1.35 | 1.28 |  |
|  |  |  |  |  |  |  |  |  |  |  |  | 2004 | 1115 | 1154 | 2269 | 149 | 177 | 327 | 1.34 | 1.53 | 1.44 |  |
|  |  |  |  |  |  |  |  |  |  |  |  | 2005 | 1121 | 1156 | 2277 | 169 | 196 | 365 | 1.51 | 1.7 | 1.6 |  |
| 32 | Loudianos G et al. | 10502776 | 1999 | Italy (Sardinia) | — | 1.7% (—441del15 mutation) | 3.4 | — | 1999 | 76 | Sardinian WD patient cohort | 1999 | — | — | ~160 | — | — | 76 | — | — | 4.75 | estimated based on Hardy—Weinberg equilibrium |
| 33 | Møller et al. | 21610751 | 2011 | Denmark | — | H1069Q:18%, W779X:16%, others 6—7% | — | — | 1960—2009 | 49 | Nationwide database | — | — | — | 550 | — | — | 49 | — | — | 0.89 |  |
| 34 | Moreno—Marro et al. | 33010964 | 2021 | Spain | — | — | — | — | 2010—2015 | 514 | Regional rare disease registry, hospital DB, orphan drug registry | 2010 | 227 | 233 | 460 | 338 | 247 | 585 | 1.49 | 1.06 | 1.28 |  |
|  |  |  |  |  |  |  |  |  |  |  |  | 2011 | 228 | 234 | 462 | 338 | 247 | 585 | 1.49 | 1.06 | 1.28 |  |
|  |  |  |  |  |  |  |  |  |  |  |  | 2012 | 228 | 234 | 462 | 369 | 262 | 631 | 1.62 | 1.12 | 1.37 |  |
|  |  |  |  |  |  |  |  |  |  |  |  | 2013 | 229 | 235 | 464 | 378 | 278 | 656 | 1.66 | 1.19 | 1.43 |  |
|  |  |  |  |  |  |  |  |  |  |  |  | 2014 | 229 | 235 | 464 | 405 | 296 | 701 | 1.77 | 1.26 | 1.52 |  |
|  |  |  |  |  |  |  |  |  |  |  |  | 2015 | 230 | 236 | 466 | 433 | 315 | 748 | 1.89 | 1.34 | 1.62 |  |
| 35 | —kayama et al. | 18424137 | 2008 | Japan | – | 1.11 | 0.073 | – | 2003—2005 | 1 child | Hokkaido 3 year old screening | 2005 | — | — | 1.1362 | 1 | 0 | 1 | — | 0 | 8.8 |  |
| 36 | Nguyen et al. | 40447697 | 2025 | Vietm | — | 1.27 | 0.06875 | — | 2022—2023 | 215 carriers | Vietmese pregant women genetic screening (8,464) | 2023 | — | — | 0.8464 | — | — | — | — | — | 1.64 | Estimated by Hardy—Weinberg |
| 37 | O'Brien et al. | 25112974 | 2014 | Ireland | 0.0187 | ~1.1 | 0.0729 | — | 1970—2011 | 50 | Nationwide multicenter registry | 1971 | 141.2 | 144.8 | 286 | — | — | 19 | — | — | 0.66 |  |
|  |  |  |  |  |  |  |  |  |  |  |  | 1979 | 150.1 | 153.9 | 304 | — | — | 22 | — | — | 0.72 |  |
|  |  |  |  |  |  |  |  |  |  |  |  | 1981 | 152.3 | 156.2 | 308.5 | — | — | 30 | — | — | 0.97 |  |
|  |  |  |  |  |  |  |  |  |  |  |  | 1986 | 155.7 | 159.6 | 315.3 | — | — | 31 | — | — | 0.98 |  |
|  |  |  |  |  |  |  |  |  |  |  |  | 1991 | 160.2 | 163.9 | 324.1 | — | — | 35 | — | — | 1.08 |  |
|  |  |  |  |  |  |  |  |  |  |  |  | 1996 | 168.4 | 171.1 | 339.5 | — | — | 40 | — | — | 1.18 |  |
|  |  |  |  |  |  |  |  |  |  |  |  | 2002 | 185.3 | 187.2 | 372.5 | — | — | 39 | — | — | 1.05 |  |
|  |  |  |  |  |  |  |  |  |  |  |  | 2006 | 203.4 | 204.7 | 408.1 | — | — | 39 | — | — | 0.96 |  |
| 38 | Paradisi et al. | 25497208 | 2015 | Venezuela | — | — | 1:152 (national), 1:84 (Sulia state) | — | 1985—2013 | 35 | Venezuelan Institute of Scientific Research (IVIC) | 2013 | — | — | — | 13 | 15 | 35 | — | — | — |  |
| 39 | Patel, Rustgi et al. | 37809179 | 2023 | USA | 0.13 | — | — | — | 2007—2017 | 3,543 cases/year | HCUPNANIS (20% sample) | 2017 | 159.1 | 166.2 | 325.3 | 1,573 | 1,970 | 3,543 | 0.99 | 1.19 | 1.09 |  |
| 40 | Poujois, Woimant et al. | 28648494 | 2018 | France | — | — | — | — | 2013 | 906 | French national Health Insurance DB (Sniiram) | 2013 | 29 | 29 | 58 | 462 | 444 | 906 | 1.65 | 1.44 | 1.5 |  |
| 41 | Reilly, Daly, Hutchinson | 8459248 | 1993 | Republic of Ireland | – | 0.36—0.41 | 1:122—1:139 | 23.10% | 1970—1989 | 26 | HIPE system, clinical records | 1986 | — | — | — | — | — | 26 | — | — | 3.6 |  |
| 43 | Sieloff EM et al. | 33747525 | 2021 | USA | — | — | — | — | 2002—2014 | 19,206 | US National Inpatient Sample | — | — | — | — | 9,923 | 9,283 | 19,206 | — | — | 0.63 |  |
| 44 | Sipilä J.O.T. et al. | 32618023 | 2020 | Finland | 0.016 | — | — | 32% | 1998—2017 | 33 | Finnish Medical Registry | 2017 | — | — | — | 15 | 18 | 33 | — | — | 0.45 |  |
| 45 | Sousa B. et al. | 37724235 | 2023 | Portugal | 0.1 | — | — | — | 1995—2015 | 94 | Not specified | 2015 | — | — | — | 50 (53.2%) | 44 (46.8%) | 94 | — | — | 0.27 (2015), 0.29 (avg 1995—2015) |  |
| 46 | Lorente—Arencibia et al. | 34620762 | 2022 | Spain (Cary Islands) | Not directly reported | p.Leu708Pro: 1.0% (Gran Canaria), others 0.8% | Gran Canaria: 1/28, Spain mainland: 1/39 | — | 1995—2015 | — | Population genetic screening (2,330) | 2015—2020 | — | — | Gran Canaria: ~850,000; Spain mainland: ~47,000,000 | — | — | — | — | — | Gran Ca—ria: 4.3, Spain mainland: 0.7 |  |
| 47 | Nurm, Miriam | 39674827 | 2024 | Estonia | — | 0.59 (p.His1069Gln) | 0.100694444 | — | 2004—2023 | 17 | Estonian Biobank | 2023 | — | — | 1366000 | 8 | 9 | 17 | — | — | 1.24 |  |
| 48 | Own—Eium, Paravee | 39198578 | 2025 | Thailand | — | 0.0082 (strict criteria) | 1:78 (strict) | — | — | 6,291 | TH6K Genome Dataset | 2024 | — | — | 69950000 | — | — | 2,102 | — | — | 3 |  |
| 49 | —ssar et al. | 40134168 | 2025 | Israel | — | — | — | 25% (4/16) | — | 16 | Southern Israel Medical Center | 2025 | — | — | ~308,000 | 8 | 8 | 16 | — | — | 5.2 |  |
| 50 | To—Mai, Xuan—Hong et al. | 38553482 | 2024 | Vietm | — | 0.59 | 3.3 | — | 2020—2023 | 338 | One medical center in Ho Chi Minh City | 2024 | — | — | — | — | — | — | — | — | — |  |
| 51 | Verma, I.C. | 11262988 | 2000 | India | — | — | 1:26 (estimated) | — | 1986—1997 | 116 | AIIMS (Delhi) | 1986—1997 | — | — | — | 71 | 45 | 116 | — | — | 3.7 |  |
| 52 | Wijayasiri, Pramudi | 34381985 | 2021 | UK | — | — | — | — | 2011—2018 | 19 | Nottingham University Hospitals NHS Trust | 2017 | 35.43 | 35.54 | **70.97** | 7 | 4 | 11 | 1.97 (0.62—3.69) | 1.13 (0.46—3.28) | 1.55 (0.77—2.77) |  |
| 53 | Yamaguchi Y et al. | 10453195 | 1999 | Japan | — | — | — | — | 1977—1996 | 3 | Pediatric screening | — | — | — | 2.42 | — | — | 3 | — | — | 1.24 |  |
| 54 | Zappu, Antonietta et al. | 18728530 | 2008 | Sardinia | 3.69 (1/2707 live births) | 1.92 (total mutations) | 1:26 | — | 2001 | 110 | Newborn screening | — | — | — | 160 | — | — | 110 | — | — | 6.875 |  |
| 55 | Zari—, Agnese et al. | 28717664 | 2017 | Latvia | — | H1069Q: 66.9%* | — | — | 1964—2012 | 68 | Clinical and genetic testing | — | — | — | — | 34 | 34 | 68 | — | — | 4.17 |  |

**Supplementary Table 3: Summary of Clinical Characteristics and outcome of WD Patients Across Included Studies**

| **No.** | **Author** | **PMID** | **Year** | **Country** | **Study Type** | **Study Period** | **Sample Size** | **Age (years)** | **Male (%)** | **Hepatic (%)** | **Neurological (%)** | **KF Ring Positive (%)** | **Low Ceruloplasmin (%)** | **Hemolytic Anemia (%)** | **Uri—ry Copper (µg/24h)** | **Oral Treatment** | **ALF (%)** | **Deaths（%）** | **Age at Death (years)** | **Cause of Death** | **Liver Transplant** | **Death after LT** | **Follow-up Duration** |
| --- | --- | --- | --- | --- | --- | --- | --- | --- | --- | --- | --- | --- | --- | --- | --- | --- | --- | --- | --- | --- | --- | --- | --- |
| 1 | Aaraj S et al. | 34608110 | 2021 | Pakistan | Retrospective | 2010–2020 | 48 | 9.74 (5–17) | 58.3 | 62.2 | — | 56.3 | 68.8 | 52.1 | 645.8 ± 528.4 | 34 DPA + zinc; 2 switched to trientine | — | 4（8.33） | 11.5 ± 3.78 | Hepatic WD (non-adherence, 3); Neurologic WD (respiratory failure, 1) | 11（22.92） | 0 | Up to 7 years |
| 2 | Abbassi et al. | 38588792 | 2024 | Morocco | Retrospective cohort | 2008–2020 | 226 | 13± 5.1 | 53.5 | 50.4 | 36.3 | 67.9 | 89.3 | — | 673.6±629.2(hepatic), 730.8±591.0(neuro) | 207 DPA; 5 zinc acetate; 5 zinc sulfate | 24/226 (10.6%) | 72（31.86） | 15.7 ± 4.5 | Acute liver failure (20); decompensated cirrhosis (41); neurological deterioration (11) | 0（0.0） | 0 | Median 5 years |
| 3 | Abdel Ghaffar et al. | 21682854 | 2011 | Egypt | Cohort | 1992–2009 | 77 | 9.92 ± 0.37 (1–18) | 55.8 | 45.5 | 7.8 | 69.2 | 93.5 | 5.4 | 84.2 elevated (19/25) | 52 DPA; others zinc or alternatives | 6/77 (7.8) | 16（20.78） | 9 ± 3 | Acute liver failure (6); decompensated cirrhosis (7); variceal bleeding (1); sepsis (2) | — | — | 58.9 ± 6.4 months |
| 4 | Fredrik Åberg et al. | 37632157 | 2023 | Sweden | registry cohort | 2002–2020 | 151 | 26 (17–42) | 50.3 | 82.8 | 17.2 | — | — | — | — | 45 trientine; 8 DPA; 5 zinc (some combined) | — | 10（6.62） | Median 64 | WD (4); cardiovascular (2); liver cancer (1); neurologic (1); other cancer (1); lung disease (1) | 14（9.27） | 0 | Median 6.6 yrs (0.2–18.8) |
| 5 | Aksoy et al. | 36524887 | 2022 | Turkey | Retrospective observatio | 2007–2020 | 24 | 34（14–57 ) | 45.8 | 100 | 100 | — | — | — | — | — | 1/24 (4.2) | 1（4.17） | 57 | Sepsis due to pulmo—ry infection | 24（100.0） | 1 | Up to 10 years |
| 6 | Seema Alam et al. | 30888628 | 2019 | India | Cohort | 2011–2018 | 66 | 9 (3.25–18.75) | 66.7 | 100 | 9.1 | 90.9 | 71.2 | 60.6 | 944 ± 747 (ACLF) | 9 (DCLD group only) | 34/66 (51.5) | 20（30.3） | — | Liver failure; failed liver transplant listing | 7（10.61） | 0 | ≤90 days |
|  |  |  |  |  |  |  |  |  |  |  |  |  |  |  | 614 ± 515 (DCLD) |  |  |  |  |  |  |  |  |
| 7 | Altraif I et al. | 23006462 | 2012 | Saudi Arabia | Retrospective cohort | 1994–2008 | 40 | 13–49 | — | 100 | 25 | 82.9 | 92.5 | — | 284.70±74.99 | 31 | 5/40 (12.5) | 8（20.0） | — | Acute liver failure (5); neuro-hepatic deterioration (2); post-LT accident (1) | 8（20.0） | 1 | 6.3 ± 2.7 years |
| 8 | —talia R. Amaris et al. | 39597786 | 2023 | USA (UNOS) | Retrospective cohort | 2002–2020 | 209 | 28 (21–40) | 59 | 100 | — | — | — | — | — | All underwent LT | 29/209 (13.9) | — | — | — | 209（100.0） | 29 | 10 years |
| 9 | Fred K Askari et al. | 14713890 | 2003 | USA | Prospective cohort | 1980–2001 | 9 | 25（19–32 ) | 33 | 100 | 11 | 77.8 | 55.6 | — | 526.7 | Trientine + zinc | 9/9 (100) | 0（0.0） | 0 | No deaths reported | 0（0.0） | 0 | 1–14 years (mean 6.2) |
| 10 | Sema Aydoğdu et al. | 14725414 | 2004 | Turkey | Retrospective cohort | 1994–2002 | 4 | 6 | — | 100 | 0 | 100 | 100 | — | 1960 (median) | — | 4/4 (100) | 4（100.0） | — | Liver failure | 2（50.0） | 2 | 2.5 years |
| 11 | Sandra Beinhardt et al. | 24076416 | 2014 | Austria | Retrospective cohort | 1961–2013 | 229 | Hepatic: 21.2±12.0; Neuro: 28.8±12.0 | 48 | 61 | 27 | 41 | — | — | 557 ± 748 (Hepatic) | Mostly DPA | 11/229 (4.8) | 17（7.42） | — | Liver failure; post-transplant complications | 30（13.1） | 4 | ` |
|  |  |  |  |  |  |  |  |  |  |  |  |  |  |  | 625 ± 932 (Neuro) |  |  |  |  |  |  |  |  |
| 12 | S. Bellaryet al. | 8655953 | 1995 | USA | Retrospective cohort | 1981–1991 | 39 | 23 ± 1.5 | — | 56 | 23 | 92 | 11.5±1.4 (ALF); 11.8±1.7 (chronic) | — | 2319 ± 346 (ALF) | Mostly DPA | 22/39 (56.4) | 8（20.51） | Within 3 weeks post-LT | Sepsis with MODS; brainstem herniation; SAH; ventricular fibrillation | 39（100.0） | 8 | 0.25–10 years (mean 4.3) |
| 13 | Barış Z et al. | 22706740 | 2012 | Turkey | Retrospective | ~1990–2010 | 16 | 5–12 | 52.7 | 100 | — | 62.5 | — | 82.6 abnormal | — | — | 15/16 (93.8) | 15（93.75） | — | Liver failure | 1（6.25） | 0 | 1–38 days |
| 14 | Schmitt de Bem R et al. | 21552664 | 2011 | Brazil | Follow-up study | 1971–2010 | 36 | 34.6 ± 10.8 (12–63) | 44.4 | 69.5 (hepatic/mixed) | 55.6 (neuro/mixed) | 55.6 | — | 16.6 | — | 35 DPA; 2 zinc; 3 switched from DPA to zinc | 4/36 (11.1%) | 3（8.33） | — | Leukemia (1); liver disease progression | 1（2.78） | 0 | 129.9 ± 108.3 months |
| 15 | Bono W et al. | 12064213 | 2002 | Morocco | Retrospective | 1988–1999 | 21 | 17.6 ± 8 (9–37) | 38.1 | 19 | 71 | 90.4 | 100 | 4.8 | 348 | 18 DPA; 9 zinc; 6 combi—tion | — | 6（28.57） | — | Infection (4); decompensated cirrhosis (2) | 0（0.0） | 0 | 0.5–17 years (median 6) |
| 16 | Chen CL et al. | 8743927 | 1996 | China(Taiwan) | Single-center retrospective | 1984–1994 | 7 | 16.2 (2–25) | — | 100 | — | — | — | — | — | — | 1/7 (14.3) | 1（14.29） | 28 days post-LT | Primary graft failure | 7（100.0） | 1 | Up to 10 years |
| 17 | Cheung et al. | 29209112 | 2017 | China(hong kong) | Retrospective cohort | 2000–2016 | 211 | 27.2 (17.1–38.6) | 49.3 | 83.4 | 14.2 | — | — | 3.8 | — | 129 DPA; 22 trientine; 42 zinc; 24 combined | 9/211 (4.3) | 26（12.32） | — | Liver failure/cirrhosis complications (76.9%) | 24（11.37） | — | 5–14 years (median 8.0) |
| 18 | Choe et al. | 32820224 | 2020 | South Korea | Population cohort | 2010–2016 | 1333 | 26.1 ± 17.2 | 57.6 | 74.1 | 38.3 | — | — | — | — | 465 treated: DPA 9.3%, zinc 3.6%, trientine 5.7%, combo 16.4% | 291/1333 (21.8) | 52（3.9） | 44.5 ± 19.3 | Liver failure/cirrhosis-related | 56（4.2） | 3 | 7 years |
| 19 | Członkowska A et al. | 36427277 | 2022 | Poland | Retrospective cohort | 1950s–2019 | 929 | 27 ± 11 | 51.3 | 44 (2010s) | 35 (2010s) | 58.5 (2010s) | — | — | — | DPA: 46.1%; zinc: 50.9% | — | 102（10.98） | — | Mainly liver failure | 45（4.84） | — | 10 years |
| 20 | Członkowska A et al. | 15742108 | 2005 | Poland | Prospective cohort | 1992–2003 | 164 | 24.8 | 55.5 | 37.2 | 42.1 | — | — | — | — | 79 DPA; 81 zinc | — | 20（12.2） | 12–46 (range) | Liver failure (10); infection (6); suicide (1); liver cancer (1); accident (1) | 2（1.22） | 1 | 11 years (study period) |
| 21 | Dal MB et al. | 34584582 | 2021 | Turkey | Retrospective cohort | 2006–2020 | 27 | 20.8 ± 11.1 | 59.3 | — | — | 52.0 (14/27) | 96.0 (26/27) | — | — | — | 5/27 (18.5) | 3（11.11） | — | MDR Klebsiella sepsis (1); brain death (2) | 29（107.41） | 3 (10.3%) | 6.1 ± 3.7 years |
| 22 | Daniel-Robin T et al. | 38818282 | 2024 | USA | Retrospective cohort | 2016–2021 | 5376 | 41.2 ± 19.4 | 52 | 33.4 | 36 | — | — | — | — | 885 patients: 182 DPA, 489 trientine, 407 zinc | — | 273（5.08） | 57.9 ± 16.7 |  | 121（2.25） | — | 2–5 years (54.4% ≥2 years) |
| 23 | Day J et al. | 37205947 | 2021 | UK | Retrospective cohort | 1981–2013 | 74 | 12.3 ( 9.7–14.7) | 58.1 | 86.5 | — | — | — | — | — | 40 DPA, 18 trientine, 2 zinc | 28/74 (37.8) | 10（13.51） | Median: 2.7 yrs | Acute: liver failure or transplant complications; Chronic: end-stage liver disease | 21（28.38） | 6 | 9.8 yrs (IQR 6.4–16.9) |
| 24 | Chen CL et al. | 1458566 | 1992 | China(Taiwan) | Retrospective cohort | 1984–1991 | 5 | 21.5（9–38 ) | 60 | 100 | — | — | — | — | — | — | — | 2（40.0） | 11 days – 3 years | Traffic accident (3 yrs post-LT), upper GI bleeding (11 days post-LT) | 5（100.0） | 2 (40%) | 3 months – 7 years |
| 25 | Thomas Daniel-Robin et al. | 35793759 | 2022 | France | Retrospective cohort | 2009–2019 | 1520 | 39 （22–56) | 51.8 | 48.8 | 29.8 | — | — | — | — | 665 (43.8%) received treatment | — | 205（13.49） | 61 (IQR 47–75) | — | 152（10.0） | — | 11 years |
| 26 | Devarbhavi H et al. | 31695246 | 2019 | India | Single-center retrospective | 1997–2016 | 68 | 14 ± 8.2 | 55.9 | 100 | 47.1 | 87 | 11 ± 8.5 | — | 640 ± 590 | 67 patients treated | 68/68 (100) | 49（72.06） | Median < 1 month | MOF, infections | 2（2.94） | — | 3 months |
| 27 | Devarbhavi H et al. | 24033813 | 2014 | India | Single-center retrospective | 1996–2012 | 61 | 9.7 ± 2.8 | 62.3 | 100 | 44.3 | 89 | 10.9 ± 8.6 | — | 820 ± 798 | All treated | 61/61 (100%) | 33（54.1） | Median 2 weeks–1.8 mo | MOF, sepsis | — | — | Not specified |
| 28 | Dhawan A et al. | 15776453 | 2005 | UK | Single-center retrospective | 1967–2000 | 57 | 11.9 (5.9–17.9) | 61.4 | 100 | 29.8 | 59.6 | 0.09 (0–0.82) | 30 | 33.7 (7.1–381.6) | 47 (82.5%) treated | 27/57 (47.4%) | 15（26.32） | Median < 10 days | MOF, bleeding, infections | 10（17.54） | 2 (20%) | 11.8 years (median) |
| 29 | Dhole S et al. | 26393179 | 2015 | India | Prospective cross-sectio—l | 2002–2004 | 12 | — | 58.3 | 25 | — | — | — | — | — | 4 treated | — |  | — | Liver failure, fibrosis |  | — | — |
| 30 | Di Giorgio A et al. | 30028785 | 2018 | Italy | Retrospective cohort | ~20 years | 6 | 11.9 (11.6–13.2) | 33.3 | 100 | — | — | — | — | — | 6 oral DPA | 6/6 (100%) | — | — | — | — | 0 | 6.2 years (3–11) |
| 31 | Yi Dong et al. | 33763932 | 2021 | China | Prospective cohort | 10 years | 14 | — | 100% | 100% | — | — | — | — | — | — | 2/14 (14.3%) | 0（0.0） | — | — | 2（14.29） | 0 | 65 months |
| 32 | Karoli— Dzieżyc et al. | 25327413 | 2014 | Poland | Retrospective cohort | 1957–2014 | 12 | 6–31 | — | 33.30% | 0% | 100% | — | — | 15–383 | — | 1/12 (8.3%) | 0（0.0） | — | — | 1（8.33） | 0 | — |
| 33 | Ilknur Erol et al. | 18079318 | 2008 | Turkey | Retrospective cohort | 2001–2006 | 17 | 13 ± 2.79 (8–17) | 70.60% | 100% | 0% | 76.50% | — | — | — | — | 3/17 (17.6%) | 2（11.76） | — | Intracranial hemorrhage (1 case) | 17（100.0） | 1 | 15 ± 10.3 months |
| 34 | Fang S et al. | 39261850 | 2024 | Germany | Retrospective observational | 2013–2018 | 70 | 43.4 ± 17.3 | 60% | 57% | 44% | — | — | — | — | 76% | — | 1（1.43） | — | — | — | — | 5.11 ± 1.3 years |
| 35 | Wei-Yuan Fang et al. | 34002136 | 2021 | China | Retrospective study | 2013–2019 | 41 | 10.43 ± 2.56 | 53.70% | 100% | 39% | 87.80% | 92.7% (<0.1 g/L) | 51.20% | 2133 ± 1940 | 39 patients treated | 41/41 (100%) | 3（7.32） | — | Acute liver failure | 3（7.32） | 0 | 44.6 ± 21.9 months |
| 36 | Güngör et al. | 31949416 | 2019 | Turkey | Retrospective cohort | 2006–2017 | 94 | 9.11 ± 3.20 | 57.50% | 100% | 11.70% | 100% | — | — | 854.35 ± 1114.24 | All 94 treated | 24/94 (25.5%) | — | — | — | — | — | — |
| 37 | Isa et al. | 39559689 | 2024 | Bahrain | Retrospective cohort | 2002–2024 | 8 | 13 ± 3.6 (9–21) | 50% | 75% | 50% | 25% | 85.70% | — | 1251.9±966.0 | All 8 treated | — | 2 (25%) | 13, 17 | Liver failure / multiorgan failure | 1 (12.5%) | 0 | 3–22 years (median 20.5) |
| 38 | Jagadisan et al. | 21691224 | 2012 | India | Retrospective cohort | 2000–2010 | 10 | 9.5 (3–15) | 50% | 10% | — | — | — | — | — | 7 patients treated (70%) | 5 /10(50%) | — | — | Liver failure / sepsis | 0（0.0） | — | Median 2.1 years |
| 39 | Gabriela Jiménez et al. | 19371968 | 2009 | Costa Rica | Retrospective cohort | 1992–2006 | 35 | 10 ± 2 | 69% | 69% | 2.90% | 22% | 100% | 11% | 388 | 33 (94%) treated | 11/35 (31.4%) | 6（17.14） | 10.5 ± 2.9 | ALF | 5（14.29） | 0 | — |
| 40 | Laimutis Kucinskas et al. | 18855987 | 2008 | Lithuania | Retrospective cohort | 1999–2008 | 13 | 26.4 ± 7.1 | 23.10% | 100% | 15.40% | 46.20% | 92.30% | 23.10% | 158.6 ± 87.2 | 11 treated | 3/13 (23.1%) | 2（15.38） | 19, 34 | Acute liver failure | — | — | — |
| 41 | Madhan Kumar et al. | 36211139 | 2022 | India | Retrospective study | 2010–2020 | 122 | 10.2 ± 1.8 | Male-to-female 3.5:1 | 7.40% | 100% (neuro) | 100% | 96.30% | — | 81.5% elevated | 27 (DPA) | — | 1（0.82） | — | Pneumonia | 0（0.0） | 0 | Median 13.5 months (3–72) |
| 42 | Haselwanter P et al. | 40419847 | 2025 | Austria | Single-center retrospective | 2012–2024 | 4 | 44 (22–56) | 75% | 100% | — | — | — | — | — | 4 | 4/4 (100%) | 1（25.0） | 22 | Multiple organ failure | 3（75.0） | 0 | ICU to discharge/death |
| 43 | Jingjing Li et al. | 40200352 | 2025 | China | Retrospective observational | 2016–2022 | 2,634 | 24.1 ± 13.0 | 55.70% | — | — | — | — | — | — | — | — | 11（0.42） | — | — | — | — | 7 years |
| 44 | na Li et al. | 34144249 | 2021 | USA | Retrospective cohort | 2006–2011 | 9,046 | 50.4 ± 0.6 | 45.80% | 1.09% ALF (main dx) | — | — | — | — | — | — | — | 233 (2.58) | — | Liver-related (30.2%), infections (18.6%) | — | — | 6 years |
| 45 | Marudanayagam R et al. | 19768148 | 2009 | UK | Descriptive retrospective | 1992–2008 | 8 | — | — | 100% (all ALF) | — | — | — | — | — | — | 8/8 (100%) | 1（12.5） | — | — | 8（100.0） | 1 | 10 years |
| 46 | Møller LB et al. | 21610751 | 2011 | Denmark | Retrospective cohort | 1960–2009 | 49 | 9–39 | 55.10% | 57.10% | 34.70% | 63.4% (26/41) | 90.2% (37/41) | — | — | — | 10 | 9（18.37） | 12–46 | Fulminant liver failure (4), cirrhosis complications (1), accident (2), other (2) | 8（16.33） | 1 (HCC recurrence) | Up to 2009 |
| 47 | Morioka et al. | 16212637 | 2005 | Japan | Retrospective cohort | 1990–2005 | 21 | 13.7 (1.4–16.5) | 57.10% | 14 cirrhosis, 7 ALF | — | 100% | — | — | — | — | 7 (ALF) | 4（19.05） | — | — | 21（100.0） | 1 | Median 78.7 months |
| 48 | Park, McCabe et al. | 1773964 | 1991 | Scotland | Epidemiologic retrospective | 1974–1989 | 33 | Hepatic: 14 ± 1.7, Neuro: 18 ± 1.9 | 39.40% | 36.40% | 30.30% | 83.3% (20/24) | 100% (20/20) | 7.1% (2/28) | 438.5±50.8 | 31 (DPA) | 4 | 12（36.36） | — | ALF (4), chronic liver disease (2), pneumonia (4), epilepsy/asphyxia (1), pancreatitis (1) | 1（3.03） | 1 (post-op 2 months) | 13.7 years (2–33) |
| 49 | Patel, Rustgi et al. | 37809179 | 2023 | USA | national inpatient cohort | 2007–2017 | 3,543 | 49 (35–62) | 44.40% | Cirrhosis: 14.5%, ALF: 5.0% | Ataxia 0.2%, Dystonia 0.4%, Tremor 0.6% | — | — | 73% (27/37) | — | Not specified | 5.00% | 89（2.51） | — | ALF, chronic liver disease | 187（5.28） | 2 | — |
| 50 | Pawaria, Alam et al. | 33368534 | 2021 | India | Prospective interventional | 2014–2019 | 37 | 9 (5–15) | 62.20% | 100% | 8.10% | — | — | 73% (27/37) | — | 37 (DPA) | All ALF | 11（29.73） | — | Liver failure, infections | 9（24.32） | — | 90 days |
| 51 | Penon-Portmann et al. | 32154060 | 2020 | Costa Rica | Retrospective cohort | 2010–2015 | 34 | 8.8 ± 3.6 (3.61–17) | 55.9% (19/34) | 50% (17/34) | 0% (0/34) | 8.8% (3/34) | 100% (34/34) | 8.8% (3/34) | 1200 | — | 6 (17.6%) | 1 (2.9) | 9 years 4 months | Fulminant liver failure | 2 (5.9) | 0 | — |
| 52 | Pfeiffenberger et al. | 25369181 | 2015 | Multicountry | Retrospective cohort | — | 14 | 53 (33–72) | 64.3% (9/14) | 100% (14/14) | 64.3% (9/14) | 71.4% (10/14) | — | — | — | 11 (DPA/Zinc/Trientine) | — | 5 (35.7) | — | Tumor progression / liver failure | 3 (21.4) | — | 1–26 months |
| 53 | Pop, Grama et al. | 35054127 | 2022 | Romania | Retrospective cohort | 2012–2018 | 9 | Children–Adolescents | — | 100% | — | — | Mean 7.75 ± 4.18 mg/dL | — | — | — | 9 | 3 (33.3) | — | Liver failure | 2 (22.2) | 0 | — |
| 54 | Poujois et al. | 28648494 | 2018 | France | nationwide study | 2013 | 906 | 20–39 | 51.00% | — | — | — | — | — | — | 37.2% Penicillamine | — | 28 (3.1) | — | — | 12 (1.3) | — | — |
| 55 | Saito T et al. | 3595645 | 1987 | Japan | Retrospective case analysis | 1965–1977 | 283 | 12.0 ± 5.5 | 55.80% | 28.60% | 58.00% | 82.3% (233/283) | — | 9.9% (28/283) | — | — | 62 | 0（0.0） | — | Acute liver failure (main cause) | 0（0.0） | - | — |
| 56 | Santiago R et al. | 26230903 | 2015 | France | Multicenter retrospective | — | 26 | 8.0 (0.8–13.9) | 38.5% (10/26) | 88.50% | 3.8% (1/26) | 18.2% (4/22) | 87.0% (20/23) | — | 183 (40–1530) | 26 (Zinc ± chelators) | 0 | 0（0.0） | - | - | 0（0.0） | - | 5 years (2–8) |
| 57 | Shen ZY et al. | 19080264 | 2008 | China | Retrospective cohort | 2000–2007 | 5 | Median 18 | — | 100% | — | — | — | — | — | — | 0 | 5（100.0） | 5 years 6 months (median 21.8 months) | - | 0（0.0） | - | 5 years 6 months |
| 58 | Sipilä, Jussi O T et al. | 32618023 | 2020 | Finland | nationwide retrospective | 1998–2017 (20 years) | 33 | 15.8 (3.8–48.1) | 45.5% (15/33) | 58% (10/17 newly diagnosed) | 40% (7/17 newly diagnosed) | — | — | — | — | Penicillamine (64%), Trientine (30%), Zinc (64%) | — | 8（24.24） | Mean 49.5 (SD 21.1) | Malignant tumors (4: liver, pancreas, lung, breast) | 5 (15%) | — | Median 32.3 years (IQR 22.1–55.6) |
| 59 | Sousa, Bebia— et al. | 37724235 | 2023 | Portugal | Multicenter retrospective | 1995–2015 | 94 | Median 16.6 | 53.2% (50/94) | 54.8% (51/94) | 10.7% (10/94) | 27% (24/89) | 88.3% (53/60 low) | — | 84 ( 41–377.5) | Penicillamine 76.1%, Trientine 40.9% | — | 6(6.4) | Not specified | Not detailed (no liver failure mentioned) | 23.9% (21/88) | Not specified | Mean 15.2 years |
| 60 | Sutcliffe, Robert P et al. | 12698088 | 2003 | UK | Prospective cohort | 1988–2000 | 24 | — | 100% | — | — | — | — | — |  |  | 4 (16.7%) | 4 (16.7) | Median 92 months (3–139) | MOF (post-op 24h), biliary sepsis (2 months), B-cell lymphoma (7 months) | 24 (100%) | 4 (16.7%) | Median 92 months (3–139) |
| 61 | Litwin T et al. | 26071888 | 2015 | Poland | Retrospective cohort | 2005–2009 | 143 | 33.5 ± 9.5 | 57% | — | 100% (Neuro) | — | 100% (assumed all) | — | 339.2 ± 522.3 (non-worsening), 220.0 ± 347.8 (worsening) | 143 (DPA or Zinc) | — | 3 (worsening group) | Long-term bedridden complications (infection etc.) | — | — | 2 years |  |
| 62 | Mai—rdi V et al. | 31113589 | 2018 | Uruguay | Retrospective descriptive | 2009–2017 | 6 | 18 (12–22) | 0% | 100% | — | 50% (2/4 tested) | 66.7% (4/6) | 100% (6/6) | 1379 (60–4393) | 6 (DPA) | 6 | 3 (50) | 12–22 | Multiple organ failure / infection | 3（50.0） | 1 (post-op death) | Max 78 months |
| 63 | Mohamed R, Tan CT, Wong NW | 8057991 | 1994 | Malaysia | Retrospective case analysis | 1981–1991 | 18 | 15.5（11–23 ) | 72.20% | 55.60% | 27.80% | 100% | 100% | 5.60% |  | 18 | 1 | 7（38.89） | 11–23 (mean 19.5) | Liver failure (6), esophageal variceal bleeding (1) | —（nan） | — | — |
| 64 | Munk DE | 34994677 | 2022 | Denmark | Retrospective cohort | 1992–2017 | 57 | 17.5 (3–39) | 56.8% (21/37) | 43.2% (16/37) | 37.8% (14/37) | — | — | — (2 hemolysis cases) | 343 | 36 (except 1 direct transplant) | 2 | 6（10.53） | — | 5 deaths | 2（3.51） | Median 11.7 years (31 days–25 years) |  |
| 65 | Orenstein | 37634880 | 2023 | Israel | Retrospective cohort | 2013–2018 | 6 | 8.7（5–12.5 ) | 50% | 100% | 0% | 0% | 100% | — | 202 (99–1716) | 6 | 1 | 0（0.0） | — | — | 1（16.67） | 0 | 4 years 3 months–9 years 1 month (median) |
| 66 | Richard et al. | 11209485 | 2000 | India (+Bhutan, Bangladesh) | Retrospective cohort | — | 60 | 18.8 ± 10.8 (5–52) | 63% | 60% | 40% | 88% | — (median ceruloplasmin 18 U/L) | — | 419 | 60 (100%) | — | 13 (21.7) | — | Fulminant liver failure (1), SBP+sepsis (4), acute renal failure (1) | 0（0.0） | 0 | Mean 108.1 ± 10.31 months |
| 67 | Svetel M, Pekmezović T et al. | 19473354 | 2009 | Serbia | Prospective cohort (part retrospective) | 1980–2007 | 142 | 23.5 ± 9.0 | 54.90% | 36% | 39.70% | — | 88% | — | 256 ± 211 | 121 | 6 | 30（21.13） | — | Cirrhosis/liver failure (16.6%), variceal bleeding (13.3%), suicide (13.3%) | — | — | 11.1 ± 8.8 years |
| 68 | Tian Y, Gong GZ et al. | 26041495 | 2016 | China | Retrospective case analysis | 2006–2013 | 9 | 7 months to 22 days | 44.4% (4/9) | 100% | 0% | 100% (9/9) | 100% (9/9) | 100% (9/9) | 1777–8549 | 6 | 9 | 3 (33.3) | 11–21 | Liver failure (2), E. coli sepsis (1) | 0（0.0） | 0 | 6–54 months (survivors) |
| 69 | van Meer S et al. | 25160780 | 2015 | Netherlands | Retrospective cohort | Median 15 years (0.1–51.2) | 130 | 16 (0–43) | 50% | 55% | 9% | 59% | 82% | 19% |  | Zinc 92% / Penicillamine 69% / Trientine 14% (n=111) | — | 7（5.38） | 39 & 63 (only HCC) | HCC deaths 2, liver complications 5, transplant-related 2 | 28 (22) | 2 | Median 14.8 years (0.1–51.2) |
| 70 | Walshe JM | 23842488 | 2013 | UK | Retrospective case analysis | 1957–2000 | 22 | 12.6（7–22 ) | 27.3% (6/22) | 100% (hepatic) | 22.7% (5/22) | 100% (at diagnosis) | 100% (<20 mg/dL) | 100% (22/22) | 300–8496 | 18 (Penicillamine / Trientine) | 4 (ALF) | 8 (36.4) | 7–41 | Liver failure (6), trauma (1), variceal bleeding (1) | 1（4.55） | 0 | 3–50 years |
| 71 | Xu San-Qing et al. | 24142730 | 2013 | China | Clinical efficacy observation | 2001–2010 | 35 | 11.6 (7–17) | 68.6% (24/35) | 74.3% (26/35 hepatomegaly) | 57.1% (20/35) | 71.4% (25/35) | 97.1% (34/35) | 8.6% (3/35) | 677.31 ± 540.66 (PCA), 1995.6 ± 700.35 (DMPS peak) | 21 (oral penicillamine) | 4 (11.4%) | 2 (5.7) | 7 & 17 | Liver failure (1), encephalopathy (1) | 0（0.0） | - | 6 months–5 years |
| 72 | Zhang Wanzong et al. | 36211271 | 2022 | China | Retrospective cohort | 2001–2015 | 180 | 47.47 ± 11.25 | 54% | 100% | — | — | — | — | — | 0 | 1 | — | Portal vein thrombosis causing ascites & liver failure | 0 | — | 36 months |  |
| 73 | Zheng Zhou et al. | 35706848 | 2022 | China | Retrospective cohort | 2010–2019 | 237 | 29 (11–63) | 51.90% | 100% | 0% | 237 (all) | — | — | — | 0 | 3 | — | Pancreatic fistula, infection, early liver failure (1 each) | 0 | — | — |  |
| 74 | Zhou J | 20557474 | 2010 | Mainland China | Multicenter cohort study | 1993–2009 | 113 | 13.0 (5.8–17.8) | — | 35.40% | — | — | — | — | — | 18 | — | — | 113 cases | WD main indication for transplant, better survival than other diseases | — | — | 5 years |
| 75 | Samanta T | 20306753 | 2009 | India | Cross-sectional hospital study | 2005–2008 | 34 | 7.7 ± 2.13 (3.2–12) | 55.9% (19/34) | 50% (17/34) | 20.6% (7/34) | 32.4% (11/34) | 82.3% (28/34) | 11.8% (4/34) | 348.5 ± 164.3 (hepatic group) | All treated with zinc / penicillamine | 3 (8.8%) | 6 (17.6) | — | Liver failure | 0（0.0） | - | 6–29 months |
| 76 | Wong RJ et al. | 20535024 | 2011 | USA | Retrospective cohort | 1999–2008 | 33 | 26.8 | 39.40% | 45.5%* | — | 42.40% | 73.90% | — | 202.3 | 24 (8 penicillamine + 16 trientine) | — | 4（12.12） | — | Multi-organ failure (3), cardiogenic shock (1) | 11（33.33） | — | 5.33 years (mean) |

**Supplementary Table 4. Meta-regression results for heterogeneity in ALF, mortality, LT, and post-LT mortality**

| **Outcome** | **Covariate** | **Coefficient (β)** | **SE** | **p-value** | **Significance** |
| --- | --- | --- | --- | --- | --- |
| ALF | Year | 0.017 | 0.031 | 0.578 | ns |
|  | logN | −0.637 | 0.201 | 0.003 | ** |
|  | Region Europe | 0.124 | 0.691 | 0.858 | ns |
|  | Region Asia | 0.735 | 0.694 | 0.295 | ns |
|  | Design cohort | 0.256 | 0.744 | 0.733 | ns |
|  | Design registry | 0.000 | 0.000 | — | ns |
| Deaths | Year | −0.012 | 0.018 | 0.490 | ns |
|  | logN | −0.397 | 0.091 | <0.001 | *** |
|  | Region Europe | −0.061 | 0.354 | 0.863 | ns |
|  | Region Asia | 0.222 | 0.390 | 0.571 | ns |
|  | Design cohort | −0.269 | 0.376 | 0.476 | ns |
|  | Design registry | −0.351 | 1.142 | 0.760 | ns |
| Liver Transplant | Year | 0.006 | 0.035 | 0.853 | ns |
|  | logN | −0.478 | 0.163 | 0.005 | ** |
|  | Region Europe | 0.497 | 0.643 | 0.443 | ns |
|  | Region Asia | 0.051 | 0.721 | 0.944 | ns |
|  | Design cohort | −0.571 | 0.689 | 0.411 | ns |
|  | Design registry | −0.569 | 1.758 | 0.747 | ns |
| Death after LT | Year | −0.037 | 0.017 | 0.039 | * |
|  | logN | −0.614 | 0.118 | <0.001 | *** |
|  | Region Europe | 0.343 | 0.372 | 0.363 | ns |
|  | Region Asia | −0.254 | 0.425 | 0.554 | ns |
|  | Design cohort | −0.551 | 0.439 | 0.217 | ns |
|  | Design registry | −1.657 | 1.213 | 0.180 | ns |

Note: * p < 0.05; ** p < 0.01; *** p < 0.001; ns = not significant.

**Supplementary Table 5. Sensitivity and leave-one-out (LOO) analyses for pooled clinical outcomes**

| Outcome | Random pooled | 95% CI (low–high) | I² (%) | LOO min | LOO max | Fixed pooled | 95% CI (low–high) | N≥20 pooled | 95% CI (low–high) | k (N≥20) |
| --- | --- | --- | --- | --- | --- | --- | --- | --- | --- | --- |
| ALF | 0.262 | 0.197–0.339 | 93.458 | 0.249 | 0.277 | 0.154 | 0.145–0.164 | 0.191 | 0.135–0.262 | 33 |
| Deaths | 0.144 | 0.111–0.185 | 95.659 | 0.138 | 0.152 | 0.078 | 0.074–0.082 | 0.115 | 0.085–0.154 | 49 |
| Liver Transplant | 0.153 | 0.115–0.200 | 92.140 | 0.143 | 0.161 | 0.070 | 0.065–0.075 | 0.113 | 0.082–0.154 | 42 |
| Death after LT | 0.039 | 0.024–0.064 | 78.266 | 0.036 | 0.046 | 0.064 | 0.053–0.078 | 0.023 | 0.012–0.044 | 28 |

Note: LOO min/max = lowest and highest pooled proportions after sequential exclusion of individual studies. N≥20 pooled indicates pooled proportions after excluding studies with sample size<20. All pooled estimates were based on random-effects models (DerSimonian–Laird estimator). High I² values indicate substantial heterogeneity, which was further investigated using meta-regression (Supplementary Table 5) and visualized in Supplementary Figure S11.

**Supplementary Table 6: Summary of Quality of Life, Psychiatric, and Neurocognitive Assessments in WD Patients Across Included Studies**

| **No.** | **Authors** | **PMID** | **Year** | **Country** | **Study Design** | **Study Period** | **Sample Size** | **Age (Years)** | **Male (%)** | **Hepatic (%)** | **Neurologic (%)** | **SF-12 Mental QOL** | **SF-12 Physical QOL** | **PHQ-9 Score** | **Depression (%, Tool)** | **Cognitive Impairment (%, Tool)** | **Bipolar Disorder (%)** | **Panic Disorder (%)** | **Anxiety (%)** | **EQ-VAS** | **Fatigue (%)** | **Sleep Disorder (%)** | **Daytime Sleepiness (%)** |
| --- | --- | --- | --- | --- | --- | --- | --- | --- | --- | --- | --- | --- | --- | --- | --- | --- | --- | --- | --- | --- | --- | --- | --- |
| 1 | Camarata MA et al. | 34044196 | 2021 | Multinational | Prospective Cohort | 2017–2020 | 62 | 41 (30–56) | 58.1 | 19.7 | 80.7 | 50.1 (41.7–56.9) | 55.7 (50.0–58.4) | 3 (1–6) | 37.3(MINI-7) | 47.5(MoCA<26) | — | — | — | — | — | — | — |
| 2 | Carta MG et al. | 22646910 | 2012 | Italy | Case-Control | 2010 | 23 | 42.0 ± 12.5 | 39.1 | — | — | 33.8 ± 9.0 | — | — | 47.8 (MDD) | — | 30.40% | 8.70% | 17.3 | — | — | — | — |
| 3 | Camarata et al. | 36521682 | 2023 | US/Europe | Cross-Sectional + Retrospective | 2017–2021 | 62 | 41 (31–56) | 54.8 | — | — | 43.0 (35.3–52.8) | 56.3 (51.7–58.4) | 3 (1–7) | 17.4current MDE (MINI-7); 30.7 self-reported | 36.7 (MoCA<26) | 6.5 lifetime | 8.2 (past 6mo) | — | — | — | — | — |
| 4 | Chevalier et al. | 37386576 | 2023 | France | Cross-Sectional | 2021 | 257 | 39.3 ± 12.6 | 53.3 | 59.5 | 39.3 | — | — | — | 73.6 mild+ (BDI-II); 11.3 moderate-severe | — | — | — | — | 75.1 ± 20.7 | — | — | — |
| 5 | Coskun AK et al. | 38803830 | 2024 | US | Longitudinal Cohort | 2017–2022 | 33 | — | — | — | — | 52.9 ± 7.7 | 53.3 ± 9.7 | 3.9 ± 4.4 | — | — | — | — | — | — | — | — | — |
| 6 | Day J et al. | 37205947 | 2021 | UK | Retrospective Cohort | 1981–2013 | 74 | 12.3 (IQR 9.7–14.7) | 58.1 | 86.5 | — | — | — | — | 49.3 (depression/anxiety/psychosis) | 14.5 (memory/academic) | — | — | — | — | — | — | — |
| 7 | Göktaş & Yalcin | 38454433 | 2024 | Turkey | Prospective Cohort | 2022–2023 | 30 | 13.8 ± 3.2 | 43.3 | 96.7 | 3.3 | — | — | CDI: 7.1 ± 5.8 | Below mild threshold (CDI<15) | — | — | — | STAI: 33.3 (state), 36.9 (trait) | — | — | — | — |
| 8 | Jernajczyk et al. | 35322347 | 2022 | Poland | Prospective Observational | 2015–2018 | 19 | 28.4 ± 8.6 | 73 | 47.3 | 42.1 | — | — | — | 14 mild | — | — | — | — | — | — | — | — |
| 9 | Komal Kumar et al. | 1996697 | 2012 | India | Cross-Sectional | — | 30 | 28.0 ± 11.2 | 76.7 | 3.3 | 76.7 | WHOQOL-BREF: 3.7 ± 0.6 (physical) | — | — | — | — | — | — | — | — | — | — | — |
| 10 | Mariño et al. | 37510937 | 2023 | Spain | Cross-Sectional Observational | — | 102 | 36.1 ± 15.1 | 57.8 | 81.4 | 18.6 | — | — | — | — | — | — | — | 42.2 (EQ-5D-5L) | 80.8 ± 17.4 | — | — | — |
| 11 | Mohr I et al. | 40337099 | 2025 | Germany | Cross-Sectional Observational | 2023 | 51 | 39 ± 15.3 | 41.2 | 70.6 | 29.4 | 49.2 ± 8.5 | 52.6 ± 5.1 | 3.9 ± 4.0 | — | — | — | — | — | — | 11.7 (severe) | 3.9 (chronic) | 7.8 (significant) |
| 12 | Schaefer M et al. | 26549350 | 2016 | Germany | Cross-Sectional | — | 68 | 36.6 ± 12.9 | 43 | 56 | 10 | SF-36: 61–77 | — | 6.1 ± 5.4 | 56% | — | — | — | — | — | — | — | — |
| 13 | Svetel et al. | 21594897 | 2011 | Serbia | Cross-Sectional (QOL) | — | 60 | — | 60 | — | — | Neuro: 64.1 ± 27.5 vs Hepatic: 77.7 ± 15.8 | — | — |  |  | — | — | — | — | — | — | — |
| 14 | Wang et al. | 38481200 | 2024 | China | Cross-Sectional | 2015–2020 | 42 | 26 (18–50) | 64.3 | 35.7 | 64.3 | — | — | — | 64.3 (HAMD≥7) | 100 symptomatic (UWDRS>0) | — (MDD) | — | 59.5(HAMA≥7) | — | — | 59.5 (PSQI≥5) | 23.8 (ESS≥10) |
| 15 | Zhan et al. | 38622213 | 2024 | China | Cross-Sectional | 2020–2021 | 134 | 18–55 | 61.2 | — | — | SF-36: 71.4 ± 9.6 | — | — | 16.9 ± 7.3 (HAMD, mild) | — | — | — | 23.3 ± 10.3 (HAMA, moderate) | — | — | — | — |
| 16 | You Z et al. | 36104472 | 2023 | China | Cross-Sectional Survey | 2015–2020 | 150 | 28.0 ± 7.9 | 66 | 28 | 50.7 | — | — | — | — | — | — | — | — | — | 24 | 32 | — |

**Supplementary Table 7: Summary of Economic Burden in Wilson Disease from Included Studies**

| **No.** | **Authors** | **PMID** | **Year** | **Country** | **Study Period** | **WD Patients** | **Annual Per Capita Hospitalization Cost (USD)** | **Liver Transplant Cost (USD)** | **Annual Per Capita Outpatient Cost (USD)** | **Annual Per Capita Sick Leave Cost (USD)** | **Additional Data & Notes** |
| --- | --- | --- | --- | --- | --- | --- | --- | --- | --- | --- | --- |
| 1 | Fang S et al. | 39582269 | 2024 | France | 2010-2019 | - | $4,658 (SD $12,988) | $2,322 (SD $3,496) | $195 (SD $215) | $860 (SD $1,448) | 90.1% hospitalized ≥1x; 8.8% received transplants; 92.9% had outpatient records; 23.3% took sick leave. |
| 2 | David Uihwan Lee et al. | 38652529 | 2024 | USA | 2016-2019 | 4,725 (total) | 2016: Median $8,890 (Q1-Q3 $4,861–$15,793); 2017: $8,102 ($4,824–$31,933); 2018: $11,586 ($5,731–$19,974); 2019: $9,810 ($5,407–$19,096) | - | - | - | Total hospitalization costs: $20.9M (2016), $27.23M (2017), $24.2M (2018), $27.25M (2019). Extreme cases (≥30 days hospitalization, 1.4%) cost median $332,777. Low-income (Q1) mortality significantly higher (p<0.001). |
| 3 | Na Li et al. | 34144249 | 2021 | USA | 2006-2011 | 9,170 (total) | 2006: $12,000; 2007: $12,800; 2008: $13,600; 2009: $14,400; 2010: $15,200; 2011: $15,800 | - | - | - | Hospitalization rate: 40.6/million (2006) → 63.4/million (2011); Median stay: 7 days; Medicare paid 39.9%; Acute kidney failure costs ↑4.1x; Annual total cost (2011): $28.8M. |
| 4 | Mariño, Zoe et al. | 37510937 | 2023 | Spain | Retrospective | 102 | – | – | Direct costs: NHS $65 (H-group), Private $125 (EH-group) | Productivity loss: 7.8% (overall), 20.4% (EH-group) | Median total cost: $77/month; 6.9% needed caregivers (EH-group dominant); 3.9% hospitalized (costs NR); 7.8% ER visits; EH-group paid extra for physiotherapy ($57/session), speech therapy ($69/session). |
| 5 | Patel, Rustgi et al. | 37809179 | 2023 | USA | 2007-2017 | 3,543 | $61,648 (mean) | $53,054 (mean) | – | – | Non-transplant hospitalization: $62,132 (mean); Transplant: $53,054 (mean). |
| 6 | Rustgi, Vinod K et al. | 34559472 | 2022 | USA | 2007-2017 | 424 | $15,168 | – | $12,444 | – | Annual WD costs 1.96× non-WD chronic liver disease ($46,644 vs $23,748). |
| 7 | Sieloff EM et al. | 33747525 | 2021 | USA | 2002-2014 | 19,206 | $10,543 | – | – | – | - |

**Supplementary Table 8. Quality assessment of included studies reporting clinical outcomes using the Joanna Briggs Institute (JBI) Critical Appraisal Checklist**

| **No.** | **Author** | **PMID** | **Sample Size** | **Q1** | **Q2** | **Q3** | **Q4** | **Q5** | **Q6** | **Q7** | **Q8** | **Q9** | **JBI Score** | **Overall**  **Quality** |
| --- | --- | --- | --- | --- | --- | --- | --- | --- | --- | --- | --- | --- | --- | --- |
| 1 | Aaraj S et al. | 34608110 | 48 | No | No | No | Yes | No | Yes | Yes | Yes | Yes | 5 | Moderate |
| 2 | Abbassi et al. | 38588792 | 226 | No | Yes | Yes | Yes | Yes | Yes | Yes | Yes | Yes | 8 | High |
| 3 | Abdel Ghaffar et al. | 21682854 | 77 | No | No | No | Yes | Yes | Yes | Yes | Yes | Yes | 6 | Moderate |
| 4 | Fredrik Åberg et al. | 37632157 | 151 | Yes | Yes | Yes | Yes | Yes | Yes | Yes | Yes | Yes | 9 | High |
| 5 | Aksoy et al. | 36524887 | 24 | No | No | No | Yes | No | Yes | No | Yes | No | 3 | Low |
| 6 | Seema Alam et al. | 30888628 | 66 | No | No | No | Yes | Yes | Yes | Yes | Yes | Yes | 6 | Moderate |
| 7 | Altraif I et al. | 23006462 | 40 | No | No | No | Yes | No | Yes | Yes | Yes | Yes | 5 | Moderate |
| 8 | talia R. Amaris et al. | 39597786 | 209 | No | Yes | Yes | Yes | Yes | Yes | Yes | Yes | Yes | 8 | High |
| 9 | Fred K Askari et al. | 14713890 | 9 | No | No | No | Yes | No | Yes | No | Yes | No | 3 | Low |
| 10 | Sema Aydoğdu et al. | 14725414 | 4 | No | No | No | Yes | No | Yes | No | Yes | No | 3 | Low |
| 11 | Sandra Beinhardt et al. | 24076416 | 229 | No | Yes | Yes | Yes | Yes | Yes | Yes | Yes | Yes | 8 | High |
| 12 | S. Bellaryet al. | 8655953 | 39 | No | No | No | Yes | No | Yes | Yes | Yes | Yes | 5 | Moderate |
| 13 | Barış Z et al. | 22706740 | 16 | No | No | No | Yes | No | Yes | No | Yes | No | 3 | Low |
| 14 | Schmitt de Bem R et al. | 21552664 | 36 | No | No | No | Yes | No | Yes | Yes | Yes | Yes | 5 | Moderate |
| 15 | Bono W et al. | 12064213 | 21 | No | No | No | Yes | No | Yes | No | Yes | No | 3 | Low |
| 16 | Chen CL et al. | 8743927 | 7 | No | No | No | Yes | No | Yes | No | Yes | No | 3 | Low |
| 16 | Chen CL et al. | 8743927 | 5 | No | No | No | Yes | No | Yes | No | Yes | No | 3 | Low |
| 17 | Cheung et al. | 29209112 | 211 | Yes | Yes | Yes | Yes | Yes | Yes | Yes | Yes | Yes | 9 | High |
| 18 | Choe et al. | 32820224 | 1333 | Yes | Yes | Yes | Yes | Yes | Yes | Yes | Yes | Yes | 9 | High |
| 19 | Członkowska A et al. | 36427277 | 929 | No | Yes | Yes | Yes | Yes | Yes | Yes | Yes | Yes | 8 | High |
| 19 | Członkowska A et al. | 36427277 | 164 | No | No | Yes | Yes | Yes | Yes | Yes | Yes | Yes | 7 | High |
| 20 | Członkowska A et al. | 15742108 | 929 | No | Yes | Yes | Yes | Yes | Yes | Yes | Yes | Yes | 8 | High |
| 20 | Członkowska A et al. | 15742108 | 164 | No | No | Yes | Yes | Yes | Yes | Yes | Yes | Yes | 7 | High |
| 21 | Dal MB et al. | 34584582 | 27 | No | No | No | Yes | No | Yes | No | Yes | No | 3 | Low |
| 22 | Daniel-Robin T et al. | 38818282 | 5376 | Yes | Yes | Yes | Yes | Yes | Yes | Yes | Yes | Yes | 9 | High |
| 23 | Day J et al. | 37205947 | 74 | No | No | No | Yes | Yes | Yes | Yes | Yes | Yes | 6 | Moderate |
| 24 | Chen CL et al. | 1458566 | 7 | No | No | No | Yes | No | Yes | No | Yes | No | 3 | Low |
| 24 | Chen CL et al. | 1458566 | 5 | No | No | No | Yes | No | Yes | No | Yes | No | 3 | Low |
| 25 | Thomas et al. | 35793759 | 1520 | Yes | Yes | Yes | Yes | Yes | Yes | Yes | Yes | Yes | 9 | High |
| 26 | Devarbhavi H et al. | 31695246 | 68 | No | No | No | Yes | Yes | Yes | Yes | Yes | Yes | 6 | Moderate |
| 26 | Devarbhavi H et al. | 31695246 | 61 | No | No | No | Yes | Yes | Yes | Yes | Yes | Yes | 6 | Moderate |
| 27 | Devarbhavi H et al. | 24033813 | 68 | No | No | No | Yes | Yes | Yes | Yes | Yes | Yes | 6 | Moderate |
| 27 | Devarbhavi H et al. | 24033813 | 61 | No | No | No | Yes | Yes | Yes | Yes | Yes | Yes | 6 | Moderate |
| 28 | Dhawan A et al. | 15776453 | 57 | No | No | No | Yes | Yes | Yes | Yes | Yes | Yes | 6 | Moderate |
| 29 | Dhole S et al. | 26393179 | 12 | No | No | No | Yes | No | Yes | No | Yes | No | 3 | Low |
| 30 | Di Giorgio A et al. | 30028785 | 6 | No | No | No | Yes | No | Yes | No | Yes | No | 3 | Low |
| 31 | Yi Dong et al. | 33763932 | 14 | No | No | No | Yes | No | Yes | No | Yes | No | 3 | Low |
| 32 | Karoli Dzieżyc et al. | 25327413 | 12 | No | No | No | Yes | No | Yes | No | Yes | No | 3 | Low |
| 33 | Ilknur Erol et al. | 18079318 | 17 | No | No | No | Yes | No | Yes | No | Yes | No | 3 | Low |
| 34 | Fang S et al. | 39261850 | 70 | No | No | No | Yes | Yes | Yes | Yes | Yes | Yes | 6 | Moderate |
| 35 | Wei-Yuan Fang et al. | 34002136 | 41 | No | No | No | Yes | No | Yes | Yes | Yes | Yes | 5 | Moderate |
| 36 | Güngör et al. | 31949416 | 94 | No | No | No | Yes | Yes | Yes | Yes | Yes | Yes | 6 | Moderate |
| 37 | Isa et al. | 39559689 | 8 | No | No | No | Yes | No | Yes | No | Yes | No | 3 | Low |
| 38 | Jagadisan et al. | 21691224 | 10 | No | No | No | Yes | No | Yes | No | Yes | No | 3 | Low |
| 39 | Gabriela Jiménez et al. | 19371968 | 35 | No | No | No | Yes | No | Yes | Yes | Yes | Yes | 5 | Moderate |
| 40 | Laimutis et al. | 18855987 | 13 | No | No | No | Yes | No | Yes | No | Yes | No | 3 | Low |
| 41 | Madhan Kumar et al. | 36211139 | 122 | No | No | Yes | Yes | Yes | Yes | Yes | Yes | Yes | 7 | High |
| 42 | Haselwanter P et al. | 40419847 | 4 | No | No | No | Yes | No | Yes | No | Yes | No | 3 | Low |
| 43 | Jingjing Li et al. | 40200352 | 2634 | Yes | Yes | Yes | Yes | Yes | Yes | Yes | Yes | Yes | 9 | High |
| 44 | Li et al. | 34144249 | 9046 | Yes | Yes | Yes | Yes | Yes | Yes | Yes | Yes | Yes | 9 | High |
| 45 | Marudayagam R et al. | 19768148 | 8 | No | No | No | Yes | No | Yes | No | Yes | No | 3 | Low |
| 46 | Møller LB et al. | 21610751 | 49 | No | No | No | Yes | No | Yes | Yes | Yes | Yes | 5 | Moderate |
| 47 | Morioka et al. | 16212637 | 21 | No | No | No | Yes | No | Yes | No | Yes | No | 3 | Low |
| 48 | Park, McCabe et al. | 1773964 | 33 | No | No | No | Yes | No | Yes | Yes | Yes | Yes | 5 | Moderate |
| 49 | Patel, Rustgi et al. | 37809179 | 3543 | Yes | Yes | Yes | Yes | Yes | Yes | Yes | Yes | Yes | 9 | High |
| 50 | Pawaria, Alam et al. | 33368534 | 37 | No | No | No | Yes | No | Yes | Yes | Yes | Yes | 5 | Moderate |
| 51 | Penon-Portmann et al. | 32154060 | 34 | No | No | No | Yes | No | Yes | Yes | Yes | Yes | 5 | Moderate |
| 52 | Pfeiffenberger et al. | 25369181 | 14 | No | No | No | Yes | No | Yes | No | Yes | No | 3 | Low |
| 53 | Pop, Grama et al. | 35054127 | 9 | No | No | No | Yes | No | Yes | No | Yes | No | 3 | Low |
| 54 | Poujois et al. | 28648494 | 906 | Yes | Yes | Yes | Yes | Yes | Yes | Yes | Yes | Yes | 9 | High |
| 55 | Saito T et al. | 3595645 | 283 | No | Yes | Yes | Yes | Yes | Yes | Yes | Yes | Yes | 8 | High |
| 56 | Santiago R et al. | 26230903 | 26 | No | No | No | Yes | No | Yes | No | Yes | No | 3 | Low |
| 57 | Shen ZY et al. | 19080264 | 5 | No | No | No | Yes | No | Yes | No | Yes | No | 3 | Low |
| 58 | Sipilä, Jussi O T et al. | 32618023 | 33 | No | No | No | Yes | No | Yes | Yes | Yes | Yes | 5 | Moderate |
| 59 | Sousa, Bebia et al. | 37724235 | 94 | No | No | No | Yes | Yes | Yes | Yes | Yes | Yes | 6 | Moderate |
| 60 | Sutcliffe, et al. | 12698088 | 24 | No | No | No | Yes | No | Yes | No | Yes | No | 3 | Low |
| 61 | Litwin T et al. | 26071888 | 143 | No | No | Yes | Yes | Yes | Yes | Yes | Yes | Yes | 7 | High |
| 62 | Mairdi V et al. | 31113589 | 6 | No | No | No | Yes | No | Yes | No | Yes | No | 3 | Low |
| 63 | Mohamed R | 8057991 | 18 | No | No | No | Yes | No | Yes | No | Yes | No | 3 | Low |
| 64 | Munk DE | 34994677 | 57 | No | No | No | Yes | Yes | Yes | Yes | Yes | Yes | 6 | Moderate |
| 65 | Orenstein | 37634880 | 6 | No | No | No | Yes | No | Yes | No | Yes | No | 3 | Low |
| 66 | Richard et al. | 11209485 | 60 | No | No | No | Yes | Yes | Yes | Yes | Yes | Yes | 6 | Moderate |
| 67 | Svetel M, et al. | 19473354 | 142 | No | No | Yes | Yes | Yes | Yes | Yes | Yes | Yes | 7 | High |
| 68 | Tian Y, et al. | 26041495 | 9 | No | No | No | Yes | No | Yes | No | Yes | No | 3 | Low |
| 69 | van Meer S et al. | 25160780 | 130 | Yes | Yes | Yes | Yes | Yes | Yes | Yes | Yes | Yes | 9 | High |
| 70 | Walshe JM | 23842488 | 22 | No | No | No | Yes | No | Yes | No | Yes | No | 3 | Low |
| 71 | Xu San-Qing et al. | 24142730 | 35 | No | No | No | Yes | No | Yes | Yes | Yes | Yes | 5 | Moderate |
| 72 | Zhang Wanzong et al. | 36211271 | 180 | Yes | Yes | Yes | Yes | Yes | Yes | Yes | Yes | Yes | 9 | High |
| 73 | Zheng Zhou et al. | 35706848 | 237 | No | Yes | Yes | Yes | Yes | Yes | Yes | Yes | Yes | 8 | High |
| 74 | Zhou J | 20557474 | 113 | No | No | Yes | Yes | Yes | Yes | Yes | Yes | Yes | 7 | High |
| 75 | Samanta T | 20306753 | 34 | No | No | No | Yes | No | Yes | Yes | Yes | Yes | 5 | Moderate |
| 76 | Wong RJ et al. | 20535024 | 33 | No | No | No | Yes | No | Yes | Yes | Yes | Yes | 5 | Moderate |

Note: Q1: Was the sample frame appropriate to address the target population? Q2: Were study participants sampled in an appropriate way? Q3: Was the sample size adequate? Q4: Were the study subjects and the setting described in detail? Q5: Was the data analysis conducted with sufficient coverage of the identified sample? Q6: Were valid methods used for the identification of the condition? Q7: Was the condition measured in a standard, reliable way for all participants? Q8: Was the statistical analysis appropriate? Q9: Was the response rate adequate, and if not, was the low response rate managed appropriately? Each item was rated as “Yes” or “No”, and total scores were summed (0–9). Overall quality was classified as High (7–9 points), Moderate (4–6 points), or Low (0–3 points).

**Supplementary Table 9. Egger’s regression test for publication bias across all outcomes**

| **Outcome** | **Egger’s p-value** | **Interpretation** |
| --- | --- | --- |
| ALF | 6.9×10^-10^ | Significant asymmetry |
| Deaths | 1.1×10^-22^ | Significant asymmetry |
| Liver Transplant | 1.1×10^-18^ | Significant asymmetry |
| Death after LT | 0.0116 | Possible bias |
